# Genetic Elements Associated with the Acquired Resistome of the Gut Microbiota in a Broiler Rooster Flock in Hungary

**DOI:** 10.3390/ani16152322

**Published:** 2026-07-29

**Authors:** János Kiss, Balázs Libisch, Chioma Lilian Ozoaduche, Hedvig Fébel, Geertrui Rasschaert, Ellen Lambrecht, Marc Heyndrickx, Mónika Szabó, Tibor Keresztény, Katalin Posta, Ferenc Olasz

**Affiliations:** 1 Agribiotechnology and Precision Breeding for Food Security National Laboratory, Institute of Genetics and Biotechnology, Hungarian University of Agriculture and Life Sciences (MATE), 2100 Gödöllő, Hungary; ozoaduche.chioma.lilian@phd.uni-mate.hu (C.L.O.); szabo.monika@uni-mate.hu (M.S.); kereszteny.tibor@uni-mate.hu (T.K.); posta.katalin@uni-mate.hu (K.P.); olasz.ferenc.gyorgy@uni-mate.hu (F.O.); 2 Doctoral School of Natural Sciences, Hungarian University of Agriculture and Life Sciences (MATE), 2100 Gödöllő, Hungary; 3 Department of Obstetrics and Food Animal Medicine Clinic, University of Veterinary Medicine, 1078 Budapest, Hungary; febel.hedvig@univet.hu; 4 Flanders Research Institute for Agriculture, Fisheries and Food (ILVO), 9090 Merelbeke-Melle, Belgium; geertrui.rasschaert@ilvo.vlaanderen.be (G.R.); ellen.lambrecht@ilvo.vlaanderen.be (E.L.); marc.heyndrickx@ilvo.vlaanderen.be (M.H.); 5 Faculty of Veterinary Medicine, Ghent University, 9820 Merelbeke-Melle, Belgium

**Keywords:** broiler chicken, APEC, multidrug-resistance, IncC plasmids, food safety, *bla*_CMY_

## Abstract

Antibiotic resistance in Gram-negative bacteria poses a global health threat, and poultry farming provides an important reservoir for multidrug-resistant pathogens. Our study aimed to characterize the faecal microbiota and acquired resistome of Ross-308 roosters in Hungary. Amplicon and shotgun metagenomics revealed a faecal microbiota dominated by the Firmicutes, Bacteroidota, and Proteobacteria and a diverse faecal resistome, including *qnrB* and an *aadA1*-bearing integron. Culture-based screening of an antibiotic-free rooster yielded the MDR *Escherichia coli* strain K1G, displaying resistance also to third-generation cephalosporins and fluoroquinolones. Whole-genome sequencing classified K1G as a serotype O23:H16-ST453 avian pathogenic *E. coli* (APEC) strain featuring a set of chromosomal virulence factors (including *astA*, *hlyE*, *lpfA*, and *iss*) and three plasmids: a phage-like plasmid, a mosaic virulence plasmid (carrying *bla*_TEM-1b_, *hlyF*, *iutA*, *ompT*, *iucD*, and *cvaC*), and an IncC type 1 resistance plasmid harbouring *bla*_CMY-2_. The detection of identical or closely related ST453 *E. coli* strains also in broiler meat in Hungary highlights a potential risk of transmission to humans through the food chain. Moreover, the carriage of multiple acquired antibiotic resistance genes in *E. coli* K1G indicates that individual chickens can harbour or transmit antibiotic resistance even in the absence of direct antibiotic exposure.

## Simple Summary

Antimicrobial resistance is viewed as a global health threat, and poultry farming provides an important reservoir for antibiotic-resistant pathogens. Our study examined the gut bacterial composition and antibiotic resistance determinants in a broiler rooster flock in Hungary. The chickens had an overall healthy gut bacterial microflora dominated by beneficial bacteria that are involved in digestion and protection against disease. However, a wide range of antibiotic resistance genes have also been identified in the chickens’ gut microflora, and a multidrug-resistant *E. coli* strain called K1G was cultured from it. Although strain K1G did not carry acquired antibiotic resistance genes on its chromosome, it possessed three plasmids, and one of them carried resistance to several important antibiotics, while the other two plasmids contained genes that could help the K1G bacterium to survive, spread, and cause infections. *E. coli* strains similar to K1G have also been reportedly detected from chicken meat in Hungary, suggesting a possible route for transmission through the food chain. In summary, our findings show that poultry can act as a reservoir of antibiotic-resistant bacteria and resistance-carrying plasmids, and our study highlights the importance of monitoring antibiotic resistance in food animals in order to protect both animal and human health.

## 1. Introduction

Antibiotic resistance (AR) is an emerging global health challenge and one of the world’s most serious threats today [1,2,3,4]. Gram-negative bacteria make up the majority of the World Health Organization’s (WHO) priority list of antibiotic-resistant pathogens against which new treatments are needed. Multidrug resistance refers to the ability of bacteria to resist the effects of multiple antimicrobial agents. A bacterial isolate is considered multidrug-resistant (MDR) if it is non-susceptible to at least one agent in ≥3 antimicrobial categories [5]. The worldwide spread of MDR bacteria poses a serious threat to both public health and the environment [4,6]. The increasing prevalence of superbugs resistant to several antibiotics has significantly reduced the effectiveness of conventional treatments, demanding immediate and coordinated global action [7,8,9]. The overuse of antibiotics in poultry has been linked to a high degree of resistance in *Escherichia coli* to therapeutically significant antibiotics, such as ampicillin, chloramphenicol, tetracycline, sulfonamides, and fluoroquinolones [4,10,11].

The European Union (EU) produced an estimated 14.1 million tons of poultry meat in 2024, representing a marked increase of 6.0% compared with its production in 2023. Hungary provides about 3.5% of the EU’s total poultry production, making it a notable contributor to the EU poultry sector [12,13]. In Hungary, poultry production is the second-largest consumer of veterinary antibiotics after swine farming, accounting for 23.7% of total use [14]. The chicken’s gastrointestinal tract harbours a complex and dynamic microbial community, referred to as the gut microbiome, which mediates interactions between diets, the host’s health, and productivity, as evidenced by the growth-promoting effects of antibiotics in chickens. Beyond its functional role, this microbiome also serves as a reservoir of pathogens affecting both poultry and humans, including *Campylobacter*, *Salmonella*, and *E. coli*, and it constitutes an important source of antimicrobial resistance genes (ARGs) [15]. In the Netherlands, in a representative sample of human extended-spectrum β-lactamase (ESBL)-positive *E. coli* strains, 35% harboured ESBL genes and 19% carried ESBL genes located on plasmids that were genetically indistinguishable from those derived from poultry *E. coli*. In addition, ESBL-producing *E. coli* was detected in 94% of a representative sample of chicken meat, and 39% of this ESBL-producing *E. coli* belonged to genotypes that were also identified in human samples. These observations indicated a possibility for the transmission of MDR *E. coli* from poultry to humans, likely through the food chain [16].

In 2022, amoxicillin was the most commonly used antibiotic in broiler chickens in Hungary (1.937 tons), followed by enrofloxacin (0.865 tons) and doxycycline (0.500 tons) [14]. Antibiotics are primarily administered orally by drinking water or feed due to the high morbidity and mortality of bacterial infections. Prophylactic application and growth promotion still persist in certain countries; however, regulations on veterinary antibiotic use have been further tightened in the EU by Regulation (EU) 2019/6 on veterinary medicinal products by specifying that antibiotics should not be applied routinely nor used to compensate for poor hygiene or poor farm management; that antibiotics should not be used to promote growth or to increase yield; and that antibiotics should not be used for prophylaxis other than in exceptional cases. The selection of AR is of particular concern from a One Health perspective, given the increasing resistance to third-generation cephalosporins and colistin, as well as high levels of aminoglycoside, tetracycline, and quinolone resistance [14,17].

In the WHO’s guidelines on Integrated Surveillance of AMR, *E. coli* is considered an enteric organism that may serve as a sentinel for antimicrobial resistance in surveillance programs, because *E. coli* is a common species in both humans and livestock, and certain *E. coli* strain variants can also cause disease. *E. coli* can also serve as a reservoir of acquired ARGs that may be transferred to human and animal pathogens transiting the intestinal tract, and thus, it provides information on the flow of Gram-negative resistance traits in the food chain [18,19]. The European Food Safety Authority (EFSA) joint report with ECDC (2020–2021) categorizes resistance in indicator *E. coli* from animals, humans, and food, showing its possible use in food chain surveillance of AR across the One Health sectors [3].

Key players in the fast distribution of ARGs among bacteria via horizontal gene transfer (HGT) are mobile genetic elements (MGEs), such as bacteriophages, conjugative plasmids, mobile genomic islands (MGIs), and transposable elements [20,21,22,23,24]. In addition to the hosts’ own recombination mechanisms, which also play a significant role in the integration of genes transmitted by transduction or natural transformation [25], transposons (Tn) and integrons are primarily responsible for the gene flow between the chromosome and the conjugative or mobilizable elements (plasmids and MGIs), which then efficiently transfer ARGs even between unrelated taxa. MDR phenotypes of most pathogens are frequently conferred by large conjugative R-plasmids belonging to the IncA, IncC, IncF, IncHI, IncN, or IncX group of plasmids [26], as well as by MGIs designated as integrative conjugative elements (ICEs) or integrative mobilizable elements (IMEs) [21,27]. In these mobile elements, ARGs are generally associated with integrons and many different Tns forming large gene clusters often called the “antibiotic resistance island” (ARI) or “MDR region” [24,28]. These carry many additional cargo genes and are subject to rapid evolutionary changes. In addition, they sometimes can move as a unit on their own, such as In104 of the SGI1 family IMEs [29,30], which appears to be a derivative of a “*res*-hunter” transposon of the Tn*402* family [31,32]. The fact that IS elements, Tns, and recombinases occur in large numbers in such ARI regions suggests that these elements are vital in the movement of ARIs or their parts between transferable elements such as plasmids and MGIs. For this reason, bacterial strains that carry large numbers of Tns and plasmids [33,34] can easily become the starting point for the evolution of such islands. A thorough investigation of the association of such elements—whether through metagenomic analyses or the examination of specific strains—can provide important insights into the spread of AR and the development of multidrug resistance.

To gain further understanding about the molecular dynamics of antimicrobial resistance in broiler chicken flocks in Hungary, we set the following main objectives for our study: (1) to analyze the bacterial composition and acquired resistome of the faecal microbiota of Ross-308 rooster broilers in Hungary; (2) to characterize the acquired ARGs, virulence determinants, and MGEs of the MDR *E. coli* strain obtained by screening for faecal *E. coli* displaying high-level aminoglycoside resistance; and (3) to compare them with genetically related MDR *E. coli* isolates of human, animal, and food origins from a One Health perspective.

## 2. Materials and Methods

### 2.1. Chicken Feeding and Rearing Conditions

A flock of day-old rooster broilers (*Gallus domesticus*) was purchased from the hatchery of Gallus Poultry Breeding and Hatcheries Ltd. (Devecser, Hungary), and the flock was reared in an experimental barn in Herceghalom, Hungary. A total of four Ross-308 roosters from the purchased flock participated in the current study from one day of age to 42 days of age. Feed and water were provided ad libitum. The roosters received 110 mg of Monensin Na (Elancoban^®^ G200, Elanco Europe Ltd., Hook, UK) coccidiostat per kg of feed during starter (days 1–11) and grower (days 12–21) diets, while it was not used in the finishing phase (days 22–42). The compositions of starter, grower, and finisher diets are given in Supplementary Table S1. The roosters were kept housed together throughout the entire rearing period in an experimental barn not used for commercial purposes, where antibiotics had not been used previously. The experimental setup consisted of four identical pens, with each pen housing 31 chicks. To assess the intestinal microbiome, one chick was randomly selected from each of the four pens for sampling. Consequently, during the rearing phase, each of the four selected experimental animals was raised in a social environment, housed together with 30 cage mates within its respective pen. The chickens did not receive antibiotics during the period of our study. Their antibiotic-free status refers not only to the experimental period but also to the previous history of the breeding line/hatchery environment. In line with the regulations of the European Union and global poultry industry standards, Gallus Ltd. (Devecser, Hungary) does not use antibiotics routinely or prophylactically for parent flocks or day-old chicks. The temperature, lighting, and ventilation of the barn were regulated according to the recommendations of the breeder.

### 2.2. Intestinal Sampling and the Culturing of E. coli Isolate K1G

Faecal samples were collected by manually expressing the luminal rectal content (the rectal digesta). No other intestinal sample types were taken. The four samples designated as CSK1 to CSK4 were collected at the age of 42 days post mortem from the rectum of these four animals into sterile plastic containers using sterile sampling spoons, and the collected samples were transported to the laboratory of the Hungarian University of Agriculture and Life Sciences (MATE) on ice under anaerobic conditions using anaerobic jars supplemented with AnaeroGen 2.5 L sachets (Oxoid, Basingstoke, UK).

Sample CSK1 was selected for culture-based studies because the *sul1* gene, which forms part of the 3′-conserved sequence of many class 1 integrons, was detected in its microbiome via shotgun metagenomics (see the Results Section 3.1). Screening for gentamicin-resistant *E. coli* was applied because the WHO classifies aminoglycosides—specifically gentamicin—as critically important antimicrobials for both human and animal use. Furthermore, related antimicrobial resistance can easily disseminate through the transmission of Enterobacterales, including *E. coli* [35]. *E. coli* isolate K1G was cultured from the rectum sample CSK1 on a violet red bile glucose (VRBG) agar plate (VWR International bvba, Leuven, Belgium) containing 16 mg/L gentamicin.

### 2.3. DNA Purification from Broiler Faeces Samples and Amplicon Sequencing

Total DNA was purified from about 0.2 g of the rectum content samples CSK1-CSK4 described in Section 2.2. DNA was purified by a chemagic DNA Stool 200 mg Kit H96 (Revvity, Waltham, MA, USA) after homogenization of the samples with a sterile stainless-steel lab spoon, and it was amplified using tagged primers 16S-F and 16S-R, covering the V3–V4 region of the bacterial 16S rRNA gene (for sequence of primers, see Supplementary Table S2) [6,36,37,38]. PCRs and DNA purifications were performed according to Illumina’s demonstrated protocol (Part # 15044223 Rev. B; Illumina, San Diego, CA, USA). Briefly, 50 ng of template DNA was used for target amplification with a KAPA HiFi Hot Start Ready Mix (Roche, Basel, Switzerland) in a 25-cycle PCR1 reaction. The PCR products were purified with 0.8 volume of KAPA Pure Beads (Roche, Basel, Switzerland). Then, 10 ng of PCR1 products was used as a template in index PCRs. Indexing was performed with Nextera XT index v2 Kit (Illumina, Santa Clara, CA, USA) primers and with the KAPA HiFi Hot Start Ready Mix in an 8-cycle PCR reaction. The PCR products were purified with 0.8 volumes of KAPA Pure Beads. The libraries were quantified with the Equalbit 1× dsDNA HS Assay Kit (Vazyme Biotech, Nanjing, China) on an Infinite Pro 200 F Nano + Fluorescent Plate Reader (Tecan, Männedorf, Switzerland).

Amplicon sequencing of V3-V4 regions of the 16S rRNA gene on the Illumina MiSeq platform (Santa Clara, CA, USA) was performed by Xenovea Ltd. (Szeged, Hungary). The Galaxy FastQC tool was applied to verify the quality and quantity of the raw amplicon sequencing data. The obtained ≥180,000 Illumina 2 × 300 bp paired-end raw sequencing reads/faecal sample displayed mean per sequence quality scores of >Q38.

### 2.4. Analyses of the Faecal Microbiota Composition

The bacterial composition of faecal microbiota samples was analyzed using the NEPHELE (https://nephele.niaid.nih.gov/, accessed on 24 March 2026) QIIME2 16S Amplicon pipeline, version 0.1.6. The 2 × 300 bp paired-end reads obtained on the Illumina MiSeq platform were first merged (yielding a mean merged fragment length of 451 bp), then quality-filtered using a Minimum Phred Quality Score of >Q20, and finally dereplicated using VSEARCH [39]. The merged V3–V4 amplicon sequences were subsequently clustered at the 97% identity level to operational taxonomic units (OTUs) by the open-reference method and were classified using the full-length SILVA 138 Small Subunit rRNA reference database.

### 2.5. Shotgun Metagenomic Sequencing and Functional Analyses

Total metagenomic DNA (gDNA) was purified from the same faecal samples (CSK1-CSK4) using the chemagic DNA Stool 200 mg Kit H96 (Revvity, Waltham, MA, USA), and shotgun metagenomic sequencing on the Illumina NovaSeq 6000 platform was performed by Xenovea Ltd. (Szeged, Hungary), which provided 23.4–27.1 million 2 × 150 bp paired-end reads per faecal sample. The most frequently observed mean per-sequence quality scores were >Q38 for both forward and reverse Illumina reads, indicating high-quality base calling. Metagenomic contig assembly was conducted with the MEGAHIT de novo assembler Galaxy Version 1.2.9, designed for assembling large and complex metagenomic data. MEGAHIT assembles the data as a whole, and no pre-processing like partitioning or normalization is required [40]. The metagenomic contig assembly provided 293,594 to 417,485 contigs per sample, with a mean contig length of 884 bp and a maximal contig length of 723,414 bp. None of the metagenomic contigs contained any unidentified (ambiguous) nucleotide positions. Acquired ARGs were identified using the ABRicate tool against the ResFinder database version 3 April 2026, with the following settings: ≥90% sequence identity and ≥60% minimum coverage. Metagenomic contigs harbouring ARGs were also annotated using Prokka (v1.14.6) [41] and the RAST KBase SDK version 2.0.0 [42] servers. BLASTP (v.2.17.0) searches of translated ORFs were performed against the NCBI Protein database. Schematic diagrams of annotated contigs were generated in SnapGene Viewer version 3.2.1 (Insightful Science, Woburn, MA, USA).

### 2.6. Whole Genome Sequencing, Sequence Assembly, Annotation, and Typing of E. coli Strain K1G

*E. coli* gDNA was purified by the ZymoBIOMICS 96 MagBead DNA kit (Tustin, CA, USA), and whole-genome sequencing (WGS) was performed on an Illumina MiSeq platform by Xenovea Ltd. (Szeged, Hungary) using the NEXTFlex Rapid XP DNA Seq Kit with UDIs (PerkinElmer, Waltham, MA, USA) for library preparation, the High Sensitivity DNA1000 Kit on TapeStation 2200 (Agilent, Santa Clara, CA, USA) for wetlab final QC, and the MiSeq 600 v3 Kit (Illumina, San Diego, CA, USA) for 2 × 300 bp paired-end sequencing. Oxford Nanopore (Oxford, UK)-compatible library generation, library quality control, and sequencing of the library on the Oxford Nanopore MinION platform at >30× coverage were performed by iBioscience Ltd. (Pécs, Hungary) with the following parameters: mean read length at 2306 bp, mean read quality at 9.1, number of reads at 95,812, and total bases at 220,969,795. Long and short reads were assembled using Unicycler Version 0.5.1 at 113× genome coverage [43] and submitted to the NCBI Genomes database under BioProject ID PRJNA1212878 and accessions CP195635 (for K1G chromosome), CP195636 (for pEc_K1G_A), CP195637 (for pEc_K1G_B), and CP195638 (for pEc_K1G_C). Annotation was added by the NCBI Prokaryotic Genome Annotation Pipeline (PGAP). Information about PGAP can be found at https://www.ncbi.nlm.nih.gov/genome/annotation_prok/, last accessed on 16 July 2026.

Serotyping and the 7-gene MLST typing were performed in silico using the Center for Genomic Epidemiology (CGE) platform (https://www.genomicepidemiology.org/) [44,45] and the EnteroBase database [46].

### 2.7. Identification of Replicons, AR Genes, Virulence Factors, Conjugative Transfer Components, and IS Elements

Replicon types were identified using the CGE PlasmidFinder-2.0 server [47]. AR genes were identified using CARD database version 3 April 2026 [48], CGE ResFinder 4.6.0 [49], MobileElementFinder v1.0.3 [50], and oriTFinder2 [51] databases. Potential virulence factors were searched using CGE VirulenceFinder 2.0.5 [52,53,54]. Conjugative transfer apparatus components were searched using the oriTFinder2 database. The known ISs or their close relatives were identified by a BLASTN search in the IS Finder database [50,55] or by using MobileElementFinder v1.0.3. The shotgun metagenomic contigs of the rooster rectum content samples CSK1–CSK4 (see Section 2.2) were mapped against the complete genome of *E. coli* K1G using the CGE tool MyDbFinder 2.0.

### 2.8. Microbial Techniques, DNA Procedures, and Biochemical Tests

For conjugation and transformation, *E. coli* strains (Supplementary Table S3) [36,37,38,56,57,58,59,60] were grown at 37 °C in Luria–Bertani (LB) broth or on agar plates and were maintained at −70 °C in LB broth containing 25% glycerol. For the selection of transconjugants and transformants, antibiotics were used at a final concentration as follows: ampicillin (Ap), 150 μg/mL; chloramphenicol (Cm), 20 μg/mL; kanamycin (Km), 30 μg/mL; spectinomycin (Sp), 50 μg/mL; streptomycin (Sm), 50 μg/mL; nalidixic acid (Nal), 20 μg/mL; gentamicin (Gm), 25 μg/mL; tetracycline (Tc), 10 μg/mL; and rifampicin (Rif), 100 μg/mL. During the selection of Ec_K1G electrotransformed with pJKI888 and pGMY6, LB + 60 μg/mL Km was applied to reduce the background growth of Ec_K1G observed at 30 μg/mL.

Plasmid DNA was purified from TG1 using a QIAprep Spin Miniprep Kit (QIAGEN, Hilden, Germany). Electrocompetent Ec_ K1G cells were prepared from 20 mL of mid-log phase LB + Cm culture grown to OD_600_ ~0.5. Cells were centrifuged at 6000× *g* for 5 min at 4 °C, resuspended in 20 mL of ice-cold sterile MilliQ water, and centrifuged again at 6500× *g* for 10 min at 4 °C. This step was repeated twice; finally, the bacterial pellet was resuspended in 200 μL 10% glycerol. For electroporation, approx. 50 ng plasmid DNA was added to 40 μL of competent cells. Electroporation was carried out in 2 mm gap electroporation cuvettes using BTX Electro Cell Manipulator 600 (BTX, San Diego, CA, USA) set to 25 μF, 186 Ohm, and 12.5 kV/cm. Then, cells were incubated at 37 °C for 1 h in 1 mL of LB broth before being spread onto selective LB plates.

Colony PCRs were carried out using DreamTaq DNA polymerase (Thermo Fisher Scientific, Waltham, MA, USA) in a final volume of 15 μL. Single colonies were suspended in a 100 μL 0.9% NaCl solution, and 1 μL was used as the template. Cycling conditions were as follows: primary denaturation at 94 °C for 2 min, 35× 94 °C for 20 s, 55 °C for 20 s, and 72 °C for 30 s, followed by final synthesis at 72 °C for 5 min.

MIC Test Strips (Liofilchem S.r.l., Roseto degli Abruzzi, Teramo, Italy) and Sensititre EU Surveillance ESBL EUVSEC2 and Sensititre EU Surveillance *Salmonella/E. coli* EUVSEC3 plates (ThermoFisher, West Sussex, UK) by means of the Sensititre Vizion System (ThermoFisher, West Sussex, UK) [61] were used for minimal inhibitory concentration (MIC) testing of antibiotics, while streptomycin and levofloxacin MICs were tested only by the MIC Test Strip. Susceptibility to streptomycin was also tested by the disk diffusion method. The interpretation of the results was in accordance with the EUCAST Clinical Breakpoint Tables v. 16.0 (valid from 1 January 2026). The *E. coli* ATCC 25922 quality control strain was tested alongside isolate K1G, and the quality control strain performed as required according to the EUCAST QC Tables v. 16.0 reference document [62].

### 2.9. Mating Experiments

To test whether any of the plasmids of the K1G strain are conjugative or mobilizable, K1G was mated with *E. coli* TG1Rif, a Rif^R^ derivative of the TG1 strain [57], in a standard mating assay as described previously [38], with the exception that mating plates were incubated for 4 h at 37 °C before suspension of the bacterial lawn. In total, 5 μL of serial dilutions (1 × −10^6^ ×) was dropped onto selective LB agar plates to determine the titer of the donor (Cm), recipient (Rif), and putative transconjugant cells, which were selected based on different combinations of antibiotics (Rif + Cm, Rif + Ap, Rif + Gm, and Rif + Sp). All colonies obtained on plates selective for transconjugants were individually tested for the antibiotic resistance markers of K1G and for their lac^+^ or lac^−^ phenotype on LB + X-gal plates, where K1G (lac^+^) forms blue colonies, while TG1Rif (lac^−^) is white.

For mobilization of plasmid pEc_K1G_C, ~50 ng of plasmids pGMY6 and pJKI888 (for references, see Supplementary Table S3), expressing the FlhDC-family activators SgaDC and AcaDC, respectively, were introduced into Ec_K1G by electroporation. Transformants were selected on LB + 2 × Km plates; several colonies were individually tested for their resistance markers; the presence of newly introduced plasmids was verified by colony PCR using primers amprevXP and BXhNdtac (Supplementary Table S2), as described [38]. Three parallel Ec_K1G/pGMY6 and Ec_K1G/pJKI888 transformant colonies were grown on LB + Cm + 2 × Km and used as donors when mating with the TG1Rif recipient, as described above. Expression of SgaDC from pGMY6 was induced with 0.05 mM IPTG during mating, while expression of AcaCD from pJKI888 was driven only by leakage of the P_tac_ promoter because of the higher toxic effect of this activator. Donor and recipient cells were selected on LB + Cm + Km and LB + Rif, respectively, while transconjugants were selected on LB + Rif + Cm and LB + Rif + Sm plates. All colonies obtained on plates selective for transconjugants were individually tested for Rif^R^, the AR markers of K1G, and the lac^+/−^ phenotype, as described above. The presence of the TG1Rif strain-specific F′ plasmid in the Rif^R^, Gm^R^, Cm^R^, Ap^R^, Sm^R^, Sp^R^, Tc^R^, Nal^S^, Km^S^, and lac^−^ transconjugant colony was confirmed by colony PCR using primers poxfor37015 and poxrev37376.

For complementation of the transfer deficiency of pEc_K1G_C, a Km^R^ derivative of SGI1-C (SGI1-C^Δ5^) was conjugated into the TG1Rif/pEc_K1G_C strain using TG1Nal::SGI1-C^Δ5^/R55^ΔTn*6187*^ as the donor strain. To construct this donor strain, plasmid R55^ΔTn*6187*^ was transferred from TG2/R55^ΔTn*6187*^ to TG1Nal::SGI1-C^Δ5^ via standard mating (see above), where the TG1Nal::SGI1-C^Δ5^/R55^ΔTn*6187*^ transconjugants were selected on LB + Nal + Km + Cm. Then, this transconjugant was mated with the TG1Rif/pEc_K1G_C recipient to obtain the TG1Rif::SGI1-C^Δ5^/pEc_K1G_C strain, which was selected on LB + Rif + Km + Cm plates. In this mating, the initial ratio of the donor and the recipient was 3:1 to compensate for the lower stationer titer of the TG1Nal::SGI1-C^Δ5^/R55^ΔTn*6187*^ donor. Several SGI1-C transconjugants were individually tested for the AR markers of pEc_K1G_C and SGI1-C^Δ5^, and the presence of free circular and integrated forms of SGI1-C^Δ5^ was also confirmed by amplification of both *attP* and DRR (direct repeat right) using primer pairs LJ2-RJ2 and attsgi1rev-RJ2, respectively [37]. Mobilization of pEc_K1G_C was assayed in a standard mating of four independent TG1Rif::SGI1-C^Δ5^/pEc_K1G_C donor colonies and TG1Nal recipient colonies grown overnight in LB + Rif + Km + Cm and LB + Nal, respectively. The initial ratio of the donor and the recipient was 5:1 to compensate for the lower stationer titer of the donor strain. Donor and recipient cells were selected on LB + Rif + Cm + Km and LB + Nal, respectively, while transconjugants were selected on LB + Nal + Cm + Ap. All colonies obtained from plates selective for transconjugants were individually tested for the AR markers of pEc_K1G_C and for chromosomal markers of donor (Rif^R^) and recipient (Nal^R^) strains. The transfer rate was calculated as the ratio of the number of confirmed transconjugants per milliliter to the donor and recipient titers determined after 4 h of mating.

### 2.10. Construction of a Phylogenetic Tree of bla_CMY-2_-Positive E. coli Strains

A reference sequence alignment-based phylogeny builder (REALPHY, Swiss Institute of Bioinformatics, Basel, Switzerland) [6,63] was applied to infer a phylogenetic tree from WGS data of the following *bla*_CMY-2_-positive *E. coli* strains, with their NCBI SRA Run or EnteroBase uberstrain provided in brackets: *E. coli* strains 23169_PR_IISPV (ERR5414571), uvzsr-PT_25_00022-C06-AR_25_00002058 (ERR14963494), O23:H16 (SRR10970325), 149/1 (SRR29142580), ESBL-20/0894 (ERR14842140), PEC15_108 (ESC_YB1703AA), Liv109 (SRR9971417), 1035-14 (ERR2205869), 1047-14 (ERR2205878), M2020-10009247 (ERR14842161), M2020-10023312 (ERR14842165), ESBL-20/0267 (ERR14842496), M2020-10030996/2-E (ERR14842172), K1G (SRR28552532), 2020-10004558/1-E (ERR14842157), M2020-10005621/1-EC (ERR14842160), and M2020-10029931/2 (ERR14842171). In the analysis, all WGS assemblies were mapped to the selected reference genome of *E. coli* strain K1G via bowtie2 [6,63].

### 2.11. Statistical Considerations

ARGs were identified in metagenomic contigs using the ABRicate tool against the ResFinder database version 3 April 2026, with the following settings: ≥90% sequence identity and ≥60% minimum coverage. The comparatively high percentage of identity threshold applied (>90%) also supports correct gene identification [64] in shotgun metagenomic contigs, where 100% coverage cannot be expected for a number of target genes. On the other hand, the minimum coverage of ≥60% corresponds to the initial default coverage parameter of ResFinder [49], and it was selected to allow the detection of assembly-fragmented genes with acceptable confidence.

For searching query metagenomic contig and plasmid sequences using the BLASTN tool against the NCBI GenBank database, matches were evaluated based on their E-values (indicating the probability of the match to occur by chance in the database), the bit score, nucleotide identity (%), and query coverage (%). Since most significant matches exhibited E-values approaching zero, metagenomic contig and plasmid relatedness were primarily assessed using the nucleotide identity and alignment coverage values.

## 3. Results

### 3.1. Faecal Bacterial Composition and Acquired Resistome Analysis

**Figure 1:**
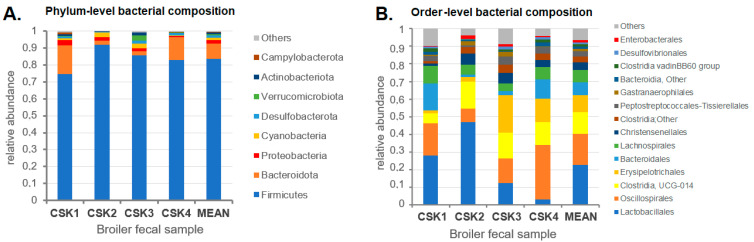
Phylum-level (**A**) and order-level (**B**) bacterial composition of broiler faeces samples CSK1–4. Relative abundances are shown on a 0 to 1 scale.

Rectum content samples designated as CSK1-CSK4 were taken from four 42-day-old birds of the same flock, and after total DNA isolation, amplicon sequencing of the 16S rRNA gene V3-V4 regions and shotgun metagenomic sequencing were performed. Bacterial composition analyses of samples CSK1-CSK4 showed that the Firmicutes dominated the faecal microbiota of all animals, with a mean relative abundance of 83.7 ± 7.1%, followed by Bacteroidota (8.7 ± 7.5%) and Proteobacteria (1.99 ± 0.98%) (Figure 1A).

Greater variability was observed among individual animals at the order level (Figure 1B), where Lactobacillales, Oscillospirales, and Clostridia UCG-014 had the highest mean relative abundance, while Enterobacterales ranged from 0.4% to 1.76%.

**Figure 2:**
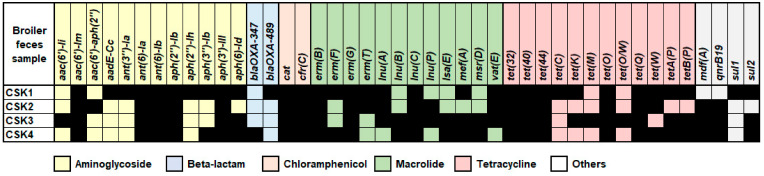
Occurrence of acquired ARGs in the microbiome of broiler faeces samples CSK1–CSK4. ARGs were identified based on ≥90% identity and ≥60% coverage against the ResFinder reference database. The presence of an ARG is indicated by a black-coloured cell.

Shotgun metagenomics revealed a high variety of acquired aminoglycoside, macrolide, tetracycline, and other ARGs in the faecal microbiome of the examined broilers (Figure 2), with the notable detection of a *qnrB* quinolone resistance determinant and a *sul1* sulfonamide resistance dihydropteroate synthase gene, indicative of the presence of a class 1 integron [65].

**Figure 3:**
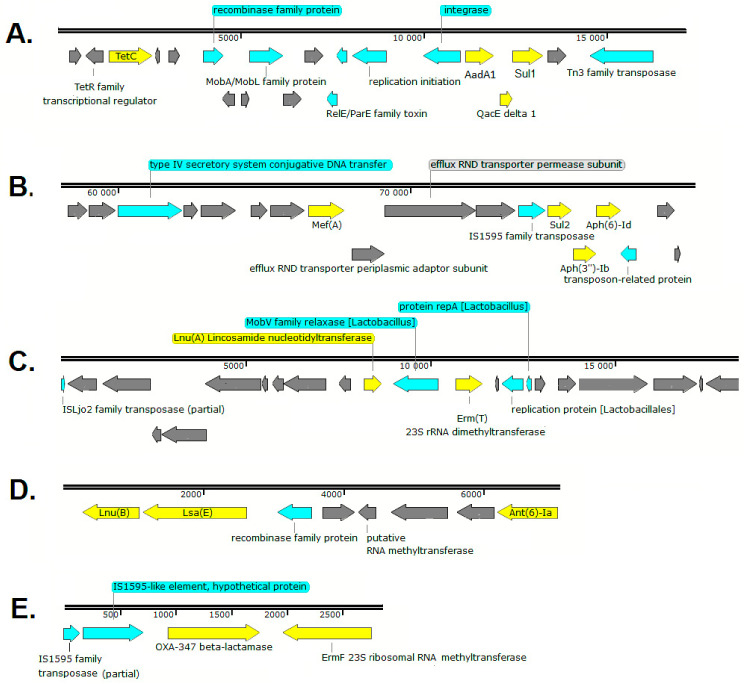
The immediate genetic context of acquired ARGs on selected broiler faecal metagenomic contigs. Subfigures (**A**), (**B**), (**C**), (**D**) and (**E**) correspond to the broiler faecal metagenomic contigs A, B, C, D and E described in Supplementary Table S4, respectively. The selected contigs were at least 2.8 kb in length and contained multiple ARGs. Light blue indicates genes that might be involved in the HGT of ARGs (plasmid-associated genes, Tns, and recombinases), and yellow colour indicates ARGs, while grey colour indicates other functions. For further information, see also Supplementary Table S4.

Selected broiler faecal metagenomic contigs with a length of at least 2.8 kb and containing multiple ARGs were individually analyzed for the immediate genetic environment of the detected ARGs and the potentially associated mobile genetic elements (Figure 3, Supplementary Table S4). Sequences homologous to metagenomic contigs A and B along their entire length were not retrieved from GenBank. Contig C showed 93% coverage at 99% identity with the *Lactobacillus johnsonii* strain UMNLJ22 chromosome (accession CP021704.1). However, the presence of genes typically found in plasmids, such as those encoding a replication initiation protein and a conjugative transfer initiation protein (relaxase) of the MobA/L family (in contig A), the conjugal transfer effector T4SS component (in contig B), and replication initiation proteins along with a MobV family relaxase (in contig C) (Figure 3A–C), suggests that these contigs and their AR genes might have derived from as-yet-uncharacterized plasmids. Contig D (Figure 3D) exhibits >99.9% homology and 99% coverage with chromosomal segments of *Streptococcus dysgalactiae*, several *Staphylococcus* spp., and *Erysipelothrix rhusiopathiae* strains, while it displays the same level of homology to plasmids described from numerous *Enterococcus faecalis* and *Listeria monocytogenes* strains, pointing to plasmid-chromosome transfer events. More than 99.7% homology was found between contig E (Figure 3E) and chromosomal regions of *Bacteroides, Sphingobacterium*, and some other *Bacteroidota* species at 97–100% coverage; however, no similar sequences were found in plasmids.

The shotgun metagenomic data included a class 1 integron in contig A from sample CSK1, harbouring an *ant(3″)-Ia* (synonym: *aadA1*) streptomycin resistance gene in its variable region (Figure 3A). The identified ARGs were flanked by a Tn*3*-related transposase in contig A, IS*1595*-family elements in contigs B and E, or an IS*Ljo2*-like element (ISL*3* family) on the *Lactobacillus* metagenomic contig C (Figure 3, Supplementary Table S4). Insertion sequences, including *IS3*, *IS4*, *IS1595*, and others, have been frequently identified across multiple MDR foodborne *Enterobacteriaceae* isolates containing composite Tns, highlighting their role in gene mobility [66,67].

### 3.2. Isolation and Characterization of E. coli Strain K1G

*E. coli* strain K1G was cultured on a VRBG agar containing 16 mg/L gentamicin from the rectum content sample CSK1, which was taken from a 42-day-old healthy Ross-308 rooster that was raised in an antibiotic-free environment and fed an antibiotic-free standard diet (Supplementary Table S1). Sample CSK1 was subjected to culture-based studies, as a *sul1* gene (which forms part of the 3′-conserved sequence of many of class 1 integrons) was detected in it by shotgun metagenomics (Figure 2 and Figure 3A, Supplementary Table S4). MICs for a range of antibiotics against strain K1G were tested using Sensititre EU Surveillance EUVSEC2 and EUVSEC3 plates and additional MIC Test Strips (Table 1), which indicated that K1G is an MDR *E. coli* strain, showing full resistance against ampicillin, cefotaxime, ceftazidime, gentamicin, ciprofloxacin, and levofloxacin according to the current EUCAST Clinical Breakpoint Tables v. 16.0 (valid from 1 January 2026), while streptomycin, tetracycline, sulfamethoxazole, nalidixic acid, and chloramphenicol were assigned to the non-wild type category by use of current EUCAST Epidemiological Cutoff (ECOFF) values for *E. coli*. Therefore, whole-genome sequencing was performed to thoroughly analyze the genetic background of this MDR phenotype, with aims (1) to investigate whether strain K1G represents a potential reservoir of clinically important ARGs within a poultry flock; (2) to examine whether these ARGs are located on the chromosome or on mobile elements like plasmids, transposons and integrons; (3) to assess whether strain K1G belongs to an internationally circulating MDR clone by determining its sequence type; and (4) to identify its virulence-associated factors.

### 3.3. Genome Sequencing of E. coli Strain K1G

The 16S rRNA gene of strain K1G, which was amplified and Sanger-sequenced using primers 27F and 1492R (Supplementary Table S2), showed 100% identity with that of *E. coli* strain LH09-a isolated from poultry faeces (GenBank CP100544) [68] and with those of other *E. coli* reference strains in GenBank. Whole-genome sequencing reads from two platforms were combined, and the assembly resulted in four circular contigs with lengths of 4,922,379 bp, 113,150 bp, 80,062 bp, and 146,632 bp, indicating that K1G harbours three plasmids. In silico serotyping and 7-gene MLST typing showed that strain K1G was a serotype O23:H16 and sequence type ST453 *E. coli* isolate, with Clermont phylotype B1. The sequence type ST453 belongs to the ST86 Complex (CC86).

### 3.4. Genomics of the K1G Chromosome

The 4.92 Mb chromosome (GC content = 50.76%) encodes 5198 annotated genes. None of the previously described acquired antibiotic resistance determinants were found on the chromosome (Supplementary Table S5), but CGE ResFinder predicted seven chromosomal genes that may confer antibiotic resistance due to point mutations. In addition, the chromosome carries genes for bacteriocin resistance and production (*mchIBCDF* and a *hlyD*-family permease). Twenty-nine virulence factors were identified by VirulenceFinder, including the heat-stable enterotoxin gene *astA*, the *mchB* microcin H47, and the *hlyE* cytolytic toxin genes. In addition, homologs of several genes involved in adhesion to host cells and/or biofilm formation were also found, such as *csgA* (curlin major subunit), *fdeC* (intimin-like adhesin), *fimH* (Type-1 fimbriae), *iha* (adhesin), *lpfA* (long polar fimbriae), *papC* (usher P fimbriae), *ychO* (encoding YchO/YchP family inverse autotransporter invasin), and *yehABCD* (encoding YHD fimbriae). Additional genes were identified that contribute to increased virulence by providing protection against environmental conditions, e.g., two copies of *gad* (surviving low pH), *fyuA*, *iha*, *irp2*, *sitA* (iron/manganese uptake), *iss/bor* (serum resistance lipoprotein), *kpsE*, *kpsMII_K23* (capsule formation), and *terC* (tellurite resistance) (see K1G_chromosome in Supplementary Table S5).

The chromosome carries 88 IS elements identified by the IS Finder database [55], 60 of which appear to be intact, and five copies of the Miniature Inverted Repeat Element MITE*Ec1*, showing 2–7 bp divergence from the canonical sequence. The 88 IS copies represent 25 different elements and belong to 11 IS families. The IS*3* family is the most dominant, with 36 copies of eight IS elements, including isoIS*2* (16 copies), IS*1203* (nine copies), and IS*Ec1*7 (six copies). The 16 isoIS*2* copies form three groups: One copy differs from IS*2* in 29 positions, ten identical copies differ in 56 positions, and the remaining five copies have an additional differing position (altogether 57) compared to IS*2*. All but two copies are delimited by 5 bp direct repeats (DR) characteristic of IS*2* insertions. One exception has a 4 bp DR, while the other has no DR. The nine IS*1203* copies are identical and differ from the canonical IS*1203* in nine positions. One of them has no DR, the others are delimited by 3 or 4 bp DRs. All isoIS*2* and IS*1203* copies appear to be intact. Apart from the truncated copy, the other five IS*Ec17* copies are intact, showing 1–3 bp differences compared to the canonical IS*Ec17*. Only two copies are bordered by 3 bp DRs. The additional five IS*3*-family elements, IS*Ec16*, IS*Ec31*, IS*Ec48*, IS*911*, and IS*Ehe3*, were found in only one mostly incomplete copy, except for IS*Ec31*, which appears to be intact and is inserted into a region assembled from some different partial IS elements.

The next most abundant family was IS*110* with 17 copies of two elements. An IS*Ec45*-like element was a single incomplete remnant copy that showed many mutations compared to the canonical IS*Ec45*, while the other 16 were IS*621*. One of them is incomplete, and one has a single-base substitution, while the others are identical to each other and the canonical IS*621*.

The IS*4* family is represented by four IS elements: one truncated and four identical intact copies of IS*4*, bordered by 12–14 bp DRs; four identical copies of IS*186*B differing in 1 bp insertion from canonical IS*186*B and bordered by 10–11 bp DRs; an IS*10*L copy with nine base substitutions and no DRs; and an incomplete IS*Cro3* copy. One intact IS*1*F copy with 8 bp DR and two inactive IS*1*H copies (one containing a frameshift mutation in the transposase gene and the other truncated) represent the IS*1* family, while one partial copy of IS*Ec10* and IS*640* represents the IS*21* family. Six copies of three mostly incomplete elements of IS*Cro1*, IS*Shdy2*, and IS*682* represent the IS*66* family. Only one of the IS*Cro1* copies and the IS*Shdy2* element appear intact and are delimited by 8 bp DRs. Both elements differ in many positions from their canonical IS sequences. The IS*256* and IS*481* families are only represented by three incomplete IS*1414* and one truncated IS*Ersp1* element, respectively.

Similarly, the IS*As1* family is also represented by possibly inactive or incomplete copies of the IS*Ec1* element. Three highly divergent (one truncated) IS*609* copies represent the IS*200*/IS*605* family. Finally, a single IS*30* copy with four divergent positions and no DR represents the IS*30* family (K1G_chromosome in Supplementary Table S5). Interestingly, IS*30*D interrupts IS*911* in *E. coli* W3110 [69] and its derivatives, such as TG1 [70], at the same position (at 334 bp) as in K1G; however, the first part of IS*911* is replaced by a partial related element, IS*Ehe3*, in K1G. Overall, it can be stated that there are several IS elements in K1G that differ from the prototype and occur in multiple copies (often delimited by DRs of a characteristic length), indicating their transposition activity.

Based on the relatively closely positioned identical elements, five putative composite transposon-like segments could be identified (Supplementary Table S5). Two IS*621* copies define a 31.2 kb element containing 23 open reading frames (ORFs), including genes for a trehalose operon, a ribonucleotide reductase operon, a *tldDE* proteolytic complex operon, a galactofuranose transport operon, and TAM transport system components. Two IS*186*B copies define the shortest Tn of 3.2 kb in length, containing a single *hokE* gene. The IS*Ec17*-based 8.7 kb Tn-like segment includes six ORFs, an α-amylase, and further uncharacterized genes. The IS*Ec1*-based 9.5 kb element contains seven ORFs, including an *rhsD* gene, a *yhh*I H-repeat protein gene, and additional uncharacterized genes. Finally, the IS*1203*-based 10.3 kb element carries a TonB-dependent receptor gene and several uncharacterized short ORFs. This Tn-like element appears to be unique, as, unlike the other four Tns, which occur in several other *E. coli* chromosomes, it is present exclusively on the K1G chromosome and was not found in any other GenBank entries. These Tn-like segments do not contain any predicted chromosomal resistance or virulence factors, except for the IS*1203*-based Tn, in which the gene encoding the TonB-dependent receptor was identified as virulence factor *iha* (encoding a bifunctional siderophore receptor and adhesin). Although the majority of these Tn-like elements occur in many sequenced *E. coli* chromosomes, there is no direct evidence for their transposition as a unit. Only the IS*186*B-based Tn is delimited by a DR, the 10 bp target duplication characteristic for IS*186* insertions, but in this Tn, both IS*186* copies are individually bordered by the same target duplication, so this does not confirm the transpositional acquisition of the entire segment, but it cannot be excluded.

In addition to the relatively large number of IS elements, the K1G chromosome also contains seven phage-derived regions. One of them (34.0 kb) is flanked by perfect DRs of 15 bp and 28 bp, which may have formed during phage integration (putative *attL/R* sites). The virulence gene *iss*/*bor* is carried by the 20.9 kb CP-933X prophage-related region. Three phage-like regions are located adjacent to tRNA synthetase genes or a tRNA gene (see K1G_chromosome in Supplementary Table S5), which is characteristic of many prophages.

### 3.5. Genomics of K1G Plasmids

#### 3.5.1. Plasmid pEc_K1G_A

One of the three large unrelated plasmids identified in the K1G genome is the 113 kb pEc_K1G_A (Supplementary Figure S1), which shows a quite different GC content (46.4%) compared to the chromosome. Its putative replication system consists of a RepB family plasmid replication initiator protein and an iteron-type *oriV* including four 21 bp imperfect DRs separated by 10, 9, and 5 bp spacing. The four iteron repeats are located downstream of the *repB* gene (160–1374 bp) in between the 1803 and 1910 bp positions. Four additional 21 bp repeats were also found between *repB* and the four-copy iterons. One of these is located immediately after the end of *repB*, while the other three form a cluster (1651–1753 bp) where the repeats are separated by 20 bp spacing. In total, 177 related plasmid sequences were found in GenBank, for which their *rep* region showed 98–100% identity to that of pEc_K1G_A. The vast majority were found in *E. coli* strains, but seven of them originated from *Shigella sonnei* strains. Using the complete plasmid sequence as a query, the database search resulted in several hundred plasmids with >50–94% coverage and >70–99% sequence identity; however, no identical plasmids were found. These relatives mostly derive from *E. coli* strains, but several *Shigella sonnei*, *Citrobacter,* and *Enterobacter* sp. plasmids were also found. Although the vast majority of the most closely related plasmids have not been assigned to Inc groups, the PlasmidFinder server classified pEc_K1G_A into the IncFIB (H89-PhagePlasmid) group, which is consistent with the fact that several related plasmids have also been identified as IncFIB replicons (e.g., pCPE35-IncFIB [CP075723.1], pIncFIB, [CP082122.1], pCPF6-IncFIB [CP075739.1]).

Genes with maintenance functions, such as partitioning and toxin-antitoxin (TA) systems were also identified on the plasmid. Interestingly, the *parA*- and *parB*-like genes are located in two independent operons: the *parA*-like AAA-family ATPase gene is followed by a putative *parD2*-like antitoxin gene, while *parB* is the first ORF of a five-gene operon including genes encoding a DNA methylase, an ABC transporter, an N-acetyltransferase, and an RGD cell attachment site-containing protein.

Three TA system-like operons consisting of two unidirectional genes preceded by a single promoter located upstream of the first ORF were found. The three systems are composed of a hypothetical protein (putative antitoxin) + YafO family toxin gene, an AbiGi-family antitoxin + a hypothetical protein (putative toxin) gene, and a TacA-family antitoxin + an AtaT-family toxin gene. As in most TA systems [71], the first ORF encodes the putative antitoxin, and the second encodes the presumptive toxin in all three operons. In addition to the TA systems, which can occur in plasmids, genomic islands, and bacteriophages as well [71,72], numerous annotated ORFs are related to phage structural genes (capsid and tail genes and non-specified phage genes). Many genes appear to be related to DNA processing, like genes for Tyrosine-type integrase, DNA polymerase I, DNA polymerase III subunits, primase, ligase, DNA methylase, helicase, and recombination exonuclease, which may also fulfil phage functions. Although a BLAST search using the full plasmid sequence retrieved hundreds of related plasmids, only five complete sequences showing >80% coverage and >97% identity to pEc_K1G_A were identified as phage isolates (CMS-2020a, GenBank: CP053388.1 and CP054387.1; 3931_30968, GenBank: OP075930.1; PhiR41_1, GenBank: PV340561.1; Cyrano, GenBank: OV696614.1). However, these also contain the plasmid-specific *repB*, *oriV*, and *par* genes (except for Cyrano, which lacks *repB-oriV*), which may be an argument against them being true phages. Based on these data, pEc_K1G_A possibly belongs to the so-called “phage-like plasmid” group, but it is not related to well-known *E. coli* phages such as P1 or P7.

pEc_K1G_A does not contain conjugation genes or *oriT*-like sequences, so it is unlikely to be capable of active HGT unless it is a phage, but this assumption requires further investigations. Unlike the chromosome and the other two plasmids, pEc_K1G_A is rather poor in IS elements, as it carries only one copy of IS*2* and IS*1203* (pEc_K1G_A in Supplementary Table S5). While IS*2* differs at two positions from the chromosomal copies, IS*1203* is identical to all copies found on the chromosome and plasmids. The low copy number of ISs and the strikingly lower GC content compared to that of the chromosome suggest that this plasmid was acquired by Ec_K1G relatively recently.

#### 3.5.2. Plasmid pEc_K1G_B

The plasmid sequence has hundreds of relatives in GenBank (>99.4% identity, 80–97% coverage), all from *E. coli* strains. The 40 kb region between 68,413 and 22,487 bp, including a 17 kb compound Tn-like segment, appears to be highly conserved (99.99%), while the region between 22,488 and 68,412 bp is much more diverse among relatives due to numerous insertions and deletions. The 86 062 bp plasmid (Supplementary Figure S2) shows a slightly lower GC content (49.5%) than the chromosome. It contains two different replication initiation protein genes: One (starting at 409 bp) encodes an IncFIB (RepB family), while the other encodes an IncFIC/IncFII (RepA1 family) replication initiator protein. The downstream region of the *repB* gene contains three iteron-like 15 bp imperfect DRs with ACYACAGCTTATATW consensus; however, their positioning is unusual as the first and second copies are separated by a relatively long (152 bp) spacing, while the space between the second and third is 33 bp. These repeats are potential candidates for serving as the *oriV* of the IncFIB replicon. Although most of *oriVs* include four or more iterons, plasmids ColE2 and ColE3 contain only two, and pSC101 has three iteron repeats [73,74], which suggests that these three 15 bp repeats may serve as iterons of *oriV*. Furthermore, both OriV-Finder and PlasAnn [75,76] predicted *oriV* in the 49,075–50,014 bp region of pEc_K1G_B, which is also supported by a BLASTN search result showing its 98% identity with the *oriV* region of *E. coli* ST131 strain EC958 plasmid pEC958 (with GenBank accession HG941719.1). The *repA1* gene is preceded by a partly overlapping leader peptide gene, *tap*, and a *copB* replication regulatory gene.

To ensure plasmid stability, pEc_K1G_B has a Type I partitioning system (*parAB*) and three TA systems: The Type I TA system includes genes *srnB* and *srnC* for a Hok/Gef family toxin and an RNA antitoxin, respectively; the two Type II systems include *yacA* antitoxin and *yacB* toxin genes and *pemI* antitoxin and *pemK* toxin genes. In addition, the plasmid has a putative post-segregational killing system, *mok/sok*.

**Figure 4:**
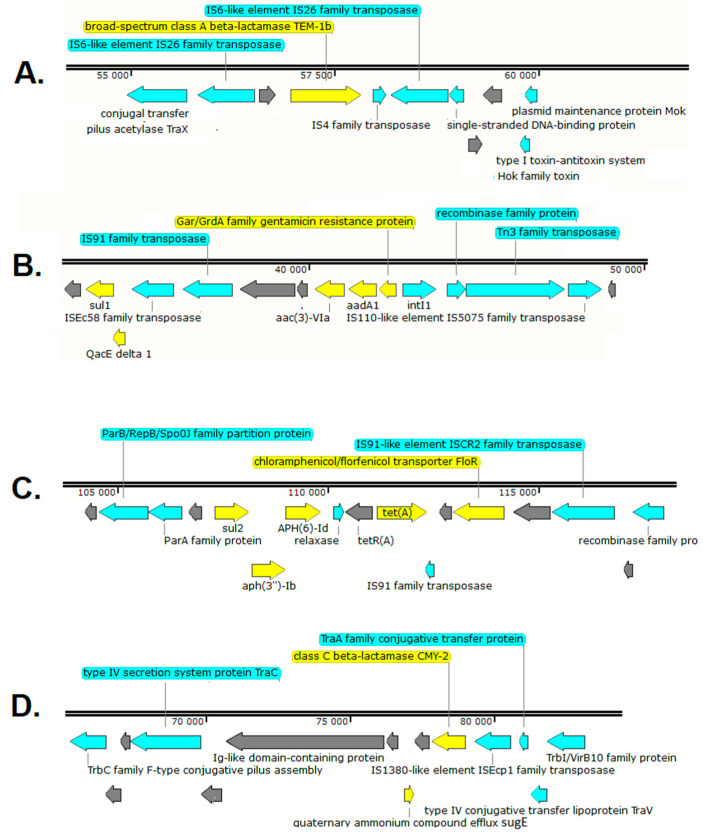
The immediate genetic context of acquired ARGs on plasmids pEc_K1G_B (**A**) and pEc_K1G_C (**B**–**D**). Colour coding is as in Figure 3. For further information, see also Supplementary Table S5.

About 31.2% of the plasmid genome is composed of mobile genetic elements, some of which are associated with resistance or virulence genes. An IS*26*-based compound Tn (55,764–58,951 bp) carries the *bla*_TEM-1b_ (Ap^R^) gene of Tn*2* (Figure 4A). Although there is no identical Tn in GenBank, there are many similar ones, most of which contain additional sequences or a short deletion between the AR cassette and one of the bordering IS*26* copies. Most are present on plasmids or chromosomes of *E. coli* strains, but some occur in *Salmonella enterica* isolates.

The next putative resistance region is embedded in an 11 kb segment of pEc_K1G_B consisting of several Tn-derived sequences and IS elements. One edge of this segment is formed by another IS*26*-based Tn (36,513–39,389 bp), which carries a partial resolvase and transposase gene deriving from a Tn*3*-family element. This compound Tn appears to be unique, as only one other plasmid was found in GenBank that carries a similar one, but its cargo sequence is 14 bp shorter at one end, while it is 23 base pairs longer at the other end than that of the Tn found in plasmid P2 (OW849318.1) of the *Klebsiella pneumoniae* isolate 147. The largest part of the 11 kb segment (39,390–45,415 bp) is derived from a Tn*21*-like transposon and contains seven genes of a *mer* module, *urf2* (EAL domain-containing protein), and the partial transposase gene of Tn*21*. The other edge of the 11 kb segment is bordered by an incomplete IS*5075* and a possibly inactive IS*1* copy.

The large Tn-like segment delimited by IS*1*A copies (67,540–84,557 bp) carries a four-gene operon for aerobactin synthesis (*iucABCD*) and a TonB-dependent siderophore receptor gene *iutA*; genes for putative transporters of H+ and fluoride ions, which may confer some degree of disinfectant/H_2_O_2_ resistance; an enolase gene; and a four-gene operon for iron and manganese uptake (*sitABCD*) that is also known as a virulence and a H_2_O_2_ resistance factor [77,78,79]. In addition, *iutA*, *iucD*, and *sitA* were also predicted as virulence genes (pEc_K1G_B in Supplementary Table S5).

The plasmid contains several genes further encoding putative resistance and virulence factors independently of the transposon-like segments. The three-gene operon of *etsABC* encodes a putative type 1 secretion system, and *cvaAB* encodes the proteins responsible for transporting and secreting the bacteriocin colicin V. A colicin operon of two genes (*cvi* and *cvaC*) encodes a colicin V immunity protein and colicin V. Furthermore, *mcmM* encoding a CPBP family intramembrane metalloprotease may contribute to the microcin resistance of Ec_K1G. Some of these genes were identified as virulence factors (*cvaC*, *etsC*, and *mchF*; pEc_K1G_B in Supplementary Table S5). Overall, based on the detection of the key APEC (Avian Pathogenic) virulence factors of *hlyF, iutA, ompT, cvaC*, and *iucD* on plasmid pEc_K1G_B and *iss* (increased serum survival/serum resistance lipoprotein Bor) on the chromosome, the K1G strain could be assigned to the APEC pathotype of *E. coli* [80,81,82].

Although pEc_K1G_B has neither an intact transfer apparatus nor a potential *oriT* sequence, it contains some genes related to conjugative functions, such as pilin acetylase gene *traX* and plasmid fertility inhibitor gene *finO*. Furthermore, the two-gene operon of *psiBA* encodes for putative SOS inhibition proteins PsiB and PsiA. PsiB has been shown to bind RecA, and it inhibits the SOS response triggered by the plasmid entry [83].

Almost a third of the plasmid is made up of mobile genetic element sequences, including numerous complete and partial IS elements. Apart from those mentioned above (IS*1*—IS*1* family, IS*5075*—IS*110* family, and IS*26*—IS*6* family), most ISs belong to the IS*3* family, which is represented by five elements: IS*2*, IS*Ec17*, IS*629*, IS*911*, and IS*1203*. One of the three IS*2* copies is interrupted by the IS*Ec17* insertion, while the other two isoIS*2* copies are intact and identical to the five chromosomal copies, but they differ in two positions from the one on pEc_K1G_A. Interestingly, the inversely oriented isoIS*2* copies are separated by only a 296 bp sequence deriving from a microcin H47 export transporter peptidase gene *mcmM* that is present on many plasmids and the chromosome of *E. coli* strain RSM044; however, this small isoIS*2*-based compound Tn-like element does not occur in any GenBank entry. Besides the incomplete IS*629* and IS*911*, there is a full IS*1203* copy, which is located near the large IS*1*-based transposon segment and identical to all other IS*1203* copies present on the chromosome and other plasmids. The IS*21*, IS*200/IS605*, and Tn*3* families are only represented by one incomplete element of IS*640*, IS*200*C, and Tn*21*, respectively. Identical IS copies define five compound Tn-like segments, but only the IS*1*-based element harbouring an Mn-transporter operon *sitABCD* and the virulence factor operon *iucABCD*+*iutA* occurs widely in *E. coli* plasmids (pEc_K1G_B in Supplementary Table S5).

#### 3.5.3. Plasmid pEc_K1G_C

The largest plasmid in Ec_K1G has a length of 146 632 bp with somewhat higher GC contents (53.5%) than that of the chromosome. The plasmid (Supplementary Figure S3) belongs to the large IncC family, which is divided into two main lineages, type 1 and type 2, based on two alleles of the *rhs1/2* and *orf1832/1847* genes and the presence or absence of two short insertions in the plasmid backbone [84]. Since the plasmid contains the type 1-specific alleles *rhs1* and *orf1832* and lacks the type 2-specific insertions I1 and I2, pEc_K1G_C can be classified as an IncC type 1 plasmid.

**Figure 5:**
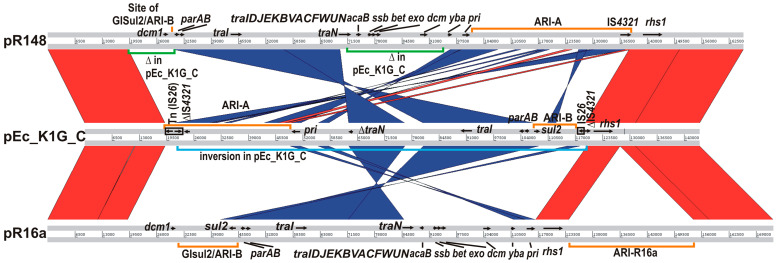
Alignment of pEc_K1G_C with plasmids pR148 (the prototype IncC Type 1 plasmid) and pR16a (containing GISul2 island, the ancestor of ARI-B). Several genes of the conserved IncC backbone (*dcm1*, *parAB*, *traI-traN*, *acaB*, *ssb bet*, *exo*, *dcm yba*, *pri*, and *rhs1*) are indicated by small horizontal arrows. The horizontal orange brackets show ARIs, the horizontal green brackets and the ∆ symbol indicate conserved backbone regions deleted from pEc_K1G_C, while the light blue bracket shows the large inverted region. IS*26* elements in plasmid pEc_K1G_C involved in generating inversion and deletion are boxed. Parts of IS4321 separated due to the IS26-mediated inversion are marked vertically as ΔIS*4321*. *ΔtraN* in plasmid pEc_K1G_C indicates the incomplete *traN* gene, which, together with the lack of *acaB* caused by transpositional deletion promoted by isoIS*Cro1* and IS*186B*, contributes to the mobilization failure of the plasmid pEc_K1G_C (see also the text). Red strips indicate homologous regions in the same orientation, while blue strips indicate homologous regions in inverted orientations between the compared plasmids. The smaller numbers indicate nucleotide positions in the concerned plasmid.

The *rep* region includes the *repA* gene and the *oriV* region containing fourteen 19 bp iteron repeats in four clusters [28]. The *repA* gene shows 99% identity with that of the IncC type 1 reference plasmid pR148 [85] and encodes a 367 aa RepA protein, which differs from that of pR148 only in one aa position. The *oriV* region shows somewhat higher divergence (94% identity, 50 differing positions) from that of pR148. The alignment of pEc_K1G_C and pR148 shows 77% coverage and 99.92% identity in the homologous regions. The plasmid possesses the largest part of the conserved IncC backbone [28], including maintenance functions, such as Type I and Type II partitioning systems *parAB* [86] and *parMRC* [87] and the *higBA* TA system [88]; the conjugative transfer apparatus, for which its genes are assembled into several operons controlled by the FlhDC family master activator AcaDC [89]; and many other conserved genes, such as *trhF*, *orf345*, *dsbC* (disulphide bond formation protein C), *orf234*, *topB* (topoisomerase III), *dcm-2* (methyltransferase), *int (xerC)* (integrase), *ter* (DNA replication terminus site-binding protein), *kfrA*, *uvrD* (helicase), *eexC* (entry exclusion protein), *acr1-acaDC* operon (AcaDC master activator and repressor), and *acr2* (repressor of *acaDC*), characteristic of IncC plasmids. However, a large inversion-deletion event has resulted in the loss of some regions and altered the characteristic order of the antibiotic resistance islands ARI-A and ARI-B. In IncC plasmids where it is present, ARI-B, which is evolved from the GISul2 island [28,90], is inserted ~14.5 kb from *higBA* at a unique position between *dcm1* and *parAB*, while ARI-A is always located 1.7 kb upstream of *rhs1* at another specific position [28]. In contrast, ARI-B, along with *parAB*, is located near *rhs1* in pEC_K1G_C, while ARI-A lies near *higBA*, and the direction and order of conserved genes are reversed between ARIs. To investigate the large-scale rearrangements in pEC_K1G_C, its sequence was compared to those of pR148 (the type 1 reference plasmid, which lacks ARI-B) and R16a, an IncC type 1 plasmid that possesses GIsul2 [91], which is the genomic island that is the ancestor of ARI-Bs and defines the standard position of ARI-B in the IncC backbone (28,932 bp of pR148). This suggested that the endpoints of the inverted region are 21,157 bp (corresponding to 137,567 bp position of pR148, which is the 885th bp of the transposase gene of IS*4321*R in *mer* side of ARI-A) and 118,005 bp (corresponding to 41,035 bp of R16a, 29th bp upstream of a resolvase gene in GISul2). Additionally, a 10,984 bp region in pR148 spanning from 17,945 bp (within ORF D745_p1024 encoding a hypothetical protein) to 28,928 bp (the insertion site of GISul2) is missing from pEc_K1G_C (Figure 5).

This deletion is characteristic of many type 1 IncC plasmids [28]. In the present constitution of pEc_K1G_C, the inverted region is bordered by an IS*26*-based compound transposon at the side of ARI-A and a single IS*26* copy at the side of ARI-B. Following the IS*26*-based Tn, ARI-A starts with the left part of the IS*4321*R copy that was originally integrated into IR_mer_, the inverted repeat next to the *mer* module of the large transposon that was the ancestor of ARI-A. The IRL of the truncated IS*4321* is joined with the inner part of IR_mer_, as is characteristic for ARI-A. At the other end of ARI-A, an IS*5075* copy is inserted into IR_tnp_, the inverted repeat next to the transposase gene of the ancient Tn, as is also characteristic for many ARI-As [28]. The missing right end of the IS*4321* located in IR_mer_ can be found next to the single IS*26* copy bordering ARI-B at the other side of the inverted segment. The IRR of this part of IS*4321* is joined to the outer part of IR_mer_.

Thus, the most likely scenario is that the ancestor of pEc_K1G_C was a “standard” type 1 plasmid with ARI-B and ARI-A at their usual positions in the plasmid backbone, i.e., ARI-B between *dcm1* and *parA* and ARI-A near the *rhs1* gene. First, the IS*26*-based transposon was inserted into the left end of ARI-B, downstream of its conserved resolvase gene, and it promoted deletion, leading to the loss of 10,984 bp backbone sequences, including *mukB*, *ftsH*, a *parB*-like gene, *dcm1*, and 13 hypothetical genes along with the left end of ARI-B, as is the case in many type 1 plasmids [28]. The next event may have been the insertion of a single IS*26* into the IS*4321* copy present at the right (*mer*) end of ARI-A, in reverse orientation to the right IS*26* copy (located adjacent to the resolvase gene of ARI-B) of the compound transposon. Finally, a homologous recombination event between the inversely oriented IS*26* copies could invert the entire region, including ARI-A and ARI-B. This latter step is supported by the fact that IS*26* ends joined to parts of the IS*4321* element are bordered by the characteristic 8 bp duplication of the target site (AATTGGCC) generated via insertion of the single IS*26* into the originally intact IS*4321* of ARI-A. Further rearrangements occurred between the conserved primase gene and *traN* possibly due to transposition events promoted by isoIS*Cro1* and IS*186*B, leading to the deletion of about 27 conserved backbone ORFs, including the 3′ part of *traN* (the last 1293 bp), *acaB*, *ssb-bet-exo*, *dcm*, and *ybaA* (Figure 5).

There is one exception (*bla*_TEM-1b_): all of the acquired ARGs responsible for the multidrug resistance of Ec_K1G are located in one of the three ARIs of pEc_K1G_C (Table 2).

ARI-A has a mosaic structure of Tn*3*-family transposon fragments disrupted by several IS and CR elements. The *tnp* end of ARI-A is assembled with a resolvase and transposase gene (*res* and *tnpA*) of the Tn*3*-like transposon and an integron containing *intI1* and three AR cassettes: a Gar/GrdA family gentamicin resistance ATP-binding protein gene, followed by *aadA1* and *aac*(3)-*VIa* (Figure 4B). Downstream of *aac*(3)*-VIa*, there are the chaperon genes *groES* and *groEL*, followed by a complex region assembled with an IS*1326*, IS*Ec58*, IS Common Region (CR) CR16, and a short segment including *qacEΔ1*, *sul1* (the 3′-conserved sequence of class 1 integrons) and a gene encoding a GNAT-family N-acetyltransferase. Finally, between the IS*1326* and the IS*4321*, there is a Tn*21*-like transposon segment characteristic of ARI-A, including three Tn genes (*urf2*, *tniA*, and *tniB*) along with a *mer* module of seven genes [92,93].

ARI-B carries *virD2*, *flo*, and *lysR* genes separated from the *tetA* and *tetR* genes by a small CR2 fragment downstream of a full CR2 copy, as is usual in IncC plasmids [28], and a partial relaxase gene (Figure 4C).

The third ARI, often called ARI-IS*Ecp1*, contains *bla*_CMY-2_, *blc* (lipocalin), and *sugE* genes associated with an IS*Ecp1* element (Figure 4D). This island is also always located in the same position in the IncC backbone, between *traA* and *orf1832* [28], but in pEc_K1G_C, it carries an additional gene encoding a LuxR family regulator, as was found in several type 1 plasmids [94]. Unlike many AR genes, no known virulence factors were found on the plasmid (pEc_K1G_C in Supplementary Table S5).

As mentioned above, pEC-K1G_C contains a number of transposable elements, most of which are involved in the assembly of ARIs. The IS and CR elements and Tn*3*-like transposon segments together account for 28.4% of the plasmid. Ten different IS elements, two CR elements, and various parts of Tn*21*-like transposons represent 10 families of mobile elements. The large IS*3* family is represented by a single IS*1203* copy, which is identical to the copies on the chromosome and the other two plasmids. The single IS*4* family element, IS*186*B, is identical to the chromosomal copies and is delimited by a 10 bp DR. Although these elements have a single base insertion in the transposase gene compared to the canonical IS*186*B or a 3 bp deletion compared to that of *IS431*, their occurrence in this plasmid and at different positions of the chromosome and the presence of 10 or 11 bp DRs (target duplication) characteristic of IS*186* insertions suggest that this variant is an actively transposing element. The IS*6* family is represented by three identical IS*26* copies, two of which border a small compound Tn-like segment in inverted orientation. Tn has no intact cargo genes, contains only a fragment of an aldehyde dehydrogenase gene and an *arsR* family regulatory gene, and occurs in hundreds of IncC plasmids, possibly causing characteristic backbone deletions starting from the left end of ARI-B [28].

As discussed earlier, IS*26* copies also seem to be responsible for the large inversion that occurred in pEc_K1G_C. The IS*21* family is represented by the IS*1326* copy integrated into the Tn*21*-derived segment of ARI-A. Two identical IS*Cro1* copies represent the IS*66* family. The elements show 50 divergent positions compared to the prototype IS*Cro1* sequence. The two copies appear to form a 10 kb compound transposon carrying three short ORFs and the N-terminal part of the IncC *traN*, which is interrupted by IS*1203*. However, the fact that this Tn-like segment does not occur in any plasmids or chromosomes in GenBank and both IS*Cro1*s are delimited by different 8 bp target duplications suggests that they were independently inserted and formed the Tn-like segment in this plasmid. Two IS*110* family elements, IS*4321* and IS*5075*, occur in this plasmid. Both are inserted into inverted repeats IR_mer_ and IR_tnp_ of the transposon ancestor of ARI-A. While IS*5075* is intact, IS*4321* is interrupted by a single IS*26* copy, and its parts are distantly located due to the inversion described earlier (Figure 5).

The IS*256* and IS*1380* families are represented by one copy of IS*Ec58* and IS*Ecp1*, respectively. IS*Ec58* is delimited by 8 bp target duplication, and its insertion separates the replication origin *oriS* from the other part of CR16. IS*Ecp1* is located in ARI-IS*Ecp1,* where it is associated with *bla*_CMY-2_. Two IS*91* family elements, CR2 and CR16, occur in pEc_K1G_C, and both are associated with ARIs. CR2 is a standard component of ARI-B, where it is usually located near *sul2*; however, in this plasmid, some resistance genes have been inserted between them. CR16, which is interrupted by IS*Ec58*, lies downstream of *groEL* in ARI-A.

The Tn*21*-like transposons of the Tn*3* family are common parts or even the precursors of ARIs [95], such as the ARI-A of IncC plasmids. In pEc_K1G_C, more than 52% of the ARI-A is derived from these elements, including a class 1 integron carrying *aadA1* and *aac(3)-VIa* cassettes (Figure 4B), which may further contribute to the rapid incorporation of resistance genes into the plasmid. Table 3 provides an overview of the main characteristics of the three plasmids of the MDR *E. coli* strain K1G.

#### 3.5.4. Mobilization of pEc_K1G_C Plasmid

Phenotyping of Ec_K1G indicated that it carries many acquired resistance determinants (Table 2), which are often located on conjugative plasmids. Therefore, a mating test was conducted to examine whether the AR genes are transferable by conjugation. Since Ec_K1G proved to be resistant to several laboratory antibiotics but not to rifampicin, we chose our Rif^R^ derivative of *E. coli* strain TG1 as the recipient, but we could not detect Cm^R^, Ap^R^, Sp^R^, or Gm^R^ transconjugants (transfer frequencies were <4.3 × 10^−10^ per donor or <2.6 × 10^−9^ per recipient cells).

After completion of the plasmid sequence assembly, this revealed that the majority of AR genes are located on the IncC family plasmid pEc_K1G_C (Table 2). Most IncC plasmids possess an effective, broad-host-range MOB_H12_ group conjugative system [96,97], which contributes to their wide dissemination among bacterial genera. Sequence comparisons of pEc_K1G_C and the fully transfer-competent type 1 plasmids pR16a and pR148 revealed that most transfer genes in pEc_K1G_C are intact; however, *traN*, encoding a mating pair stabilization protein, is partial, and the regulator gene *acaB*, coding for the activator of *acaCD* genes [98], which in turn encodes the master activator AcaCD required for the expression of many conserved IncC genes, including *tra* operons [89], is missing. Although the absence of any of these genes may explain the transfer deficiency of an IncC plasmid [98,99], all other *tra* genes, *mobI*, and the *oriT* sequence [36] of pEc_K1G_C are intact; thus, mobilization of the plasmid by complementation of the missing factors appeared to be possible.

First, the lack of the supposedly missing AcaCD activator was complemented by expression of AcaCD or the closely related SGI1-derived activator SgaDC from Km^R^ p15A-based plasmids under the control of the P_tac_ promoter (pJKI888—AcaDC; pGMY6—SgaDC, Supplementary Table S3). AcaCD and SgaDC have previously been shown to be able to substitute for each other in the activation of AcaCD-dependent promoters of IncC plasmids and SGI1 [37,59,89,100]. Plasmids expressing one of these activators were introduced into Ec_K1G by electroporation, and the resulting strains were used as donors in standard matings with the TG1Rif recipient. Even though an excess of conjugation activators was provided in trans in this setup, very few transconjugant-like colonies were obtained by Rif + Cm and Rif + Sm selection. Further phenotyping of colonies from Rif + Cm selection revealed that they were Rif^R^ mutant donors, while most colonies from Rif + Sm selection proved to be the Sm-tolerant clones of the recipient strain. However, we could identify one among the colonies obtained from mating with the Ec_K1G/pGMY6 donor, which proved to be Rif^R^ (chromosomal marker of TG1Rif recipient), Gm^R^, Cm^R^, Ap^R^, Sm^R^, Sp^R^, and Tc^R^ (AR markers of pEc_K1G_C) but not Nal^S^ (Nal^R^ is a chromosomal marker of Ec_K1G donor) and Km^S^ (Km^R^ was the marker of the complementing plasmid in the donor). Unlike the *lacZ*^+^ Ec_K1G donor, it formed a white colony on the LB + X-gal plate (*lacZ^−^*) and gave a positive PCR result using F-plasmid-specific primers, confirming the presence of the F’ plasmid of the TG1Rif recipient. Thus, it was accepted as a true pEc_K1G_C transconjugant TG1Rif clone. Nevertheless, the plasmid transfer occurred at a very low frequency (~7.1 × 10^−8^ per donor and ~1.0 × 10^−7^ per recipient cells) by the aid of the SgaDC activator, whereas, in the case of AcaCD, the transfer was completely undetectable (<9.1 × 10^−8^ per donor and <1.4 × 10^−7^ per recipient).

In the previous experiment, only the lack of AcaCD was complemented; however, TraN was also shown to be essential for plasmid transfer. SGI1 encodes not only SgaDC, which can substitute for the AcaCD activator, but also some Tra proteins, including TraN_S_, which can also substitute the plasmid-encoded TraN_C_ [37,59,99,100,101,102]. Therefore, we decided to complement the transfer deficiencies of pEc_K1G_C by introducing an SGI1 variant into TG1Rif/pEc_K1G_C. Since all resistance markers of SGI1 (ACSSuT, ref. [103]) are present on pEc_K1G_C, we chose our SGI1-C^ΔP5^ mutant containing the *aph(3′)-I* (Km^R^) gene inserted upstream of ORF S022 [58]. Although insertion of the Km^R^ gene in the promoter region of S022 reduces the mobilization efficiency of SGI1-C^ΔP5^, it was enough to transfer it into the TG1Rif/pEc_K1G_C recipient from strain TG1Nal::SGI1-C^ΔP5^/R55^ΔTn*6187*^. The SGI1-C^ΔP5^ transconjugants selected on LB + Rif + Km + Cm plates were obtained with a frequency of 1.0 × 10^−4^ per donor or 3.4 × 10^−4^ per recipient CFU/mL. The presence of free circular and integrated forms of SGI1-C^Δ5^ in transconjugants was confirmed by amplification of *attP* (the joined ends in the free plasmid-like SGI1 element) and DRR (junction region between the right end of integrated SGI1 and the chromosomal *attB* site at the end of *trmE* (Supplementary Figure S4)) [38,104]. Four independent TG1Rif::SGI1-C^Δ5^/pEc_K1G_C transconjugants were then mated with the TG1Nal recipient. Transconjugants selected on LB + Nal + Cm + Ap were obtained at low but readily detectable frequencies (3.6 × 10^−6^ per donor and 1.7 × 10^−7^ per recipient CFU/mL). Transconjugants showed the expected AR phenotype (Nal^R^, Ap^R^, Cm^R^, Sm^R^, Sp^R^, Gm^R^, Tc^R^, but Rif^S^, Km^S^), confirming the mobilization of pEc_K1G_C via the complementation of its transfer deficiency by SGI1-C^Δ5^.

### 3.6. Detection of E. coli K1G-Encoded ARGs and Virulence Genes in the Faecal Metagenome of the Examined Roosters

The shotgun metagenomic contigs of the rooster rectum content samples CSK1-CSK4 (see Section 2.2) were mapped against the complete genome of *E. coli* K1G, and metagenomic contigs displaying 100% identity with 100% coverage against the corresponding part in the *E. coli* K1G genome were further analyzed for acquired ARGs and virulence factors via the use of a coverage threshold of >20% for individual genes. A >20% coverage threshold was used here because these *E. coli* genes have already been identified with full coverage in the complete genome of strain K1G. These results revealed that, besides a number of other *E. coli* genes, *bla*_TEM-1b_, *iucB*, and *iucC* encoded by plasmid pEc_K1G_B were detected in the metagenome of the faecal sample CSK1. The *tet(A)* gene of plasmid pEc_K1G_C was identified in the metagenome of sample CSK2, while the *tet(A)*, *aph(3″)-Ib (strA)*, and *aph(6)-Id (strB)* genes of plasmid pEc_K1G_C and the *bla*_TEM-1b_ gene of pEc_K1G_B were demonstrated in the metagenome of sample CSK3. Furthermore, the *bla*_TEM-1b_ gene and *iucB* encoded by pEc_K1G_B were also detected in the metagenome of sample CSK4. These ARGs were not included in Figure 2, as these ORFs had less than 60% coverage by the encoding shotgun metagenomic contigs.

### 3.7. Serotype O23:H16 and Sequence Type ST453 E. coli Strains Reported from Hungary and Other Countries

A search for serotype O23:H16 *E. coli* strains in EnteroBase [46] provided a number of such strains from various source types and global locations (Supplementary Table S6). ST453 isolates of this serotype were recovered both from environmental samples (hospital wastewater and water/river cisterns) and human samples (such as urine), as well as from food animals, including chickens, turkey, and livestock (Supplementary Table S6). Taking a closer look at Hungary, *bla_CMY-2_*-positive ST453 *E. coli* was cultured in 2020 from both broiler caecum and broiler meat, as well as from broiler faeces in 2021, and the 2020 isolates harboured essentially the same acquired ARGs and chromosomal point mutations leading to quinolone resistance as those harboured by the 2021 strain *E. coli* K1G (Supplementary Table S7). These K1G-like ST453 *E. coli* isolates from broiler meat in Hungary, with NCBI BioProject number PRJEB70301, were submitted to NCBI SRA by the DTU National Food Institute, Technical University of Denmark (Lundtofte, Denmark, Supplementary Table S7).

### 3.8. Construction of a Phylogenetic Tree of Selected bla_CMY-2_-Positive E. coli Isolates of Human, Animal, and Food Origin

**Figure 6:**
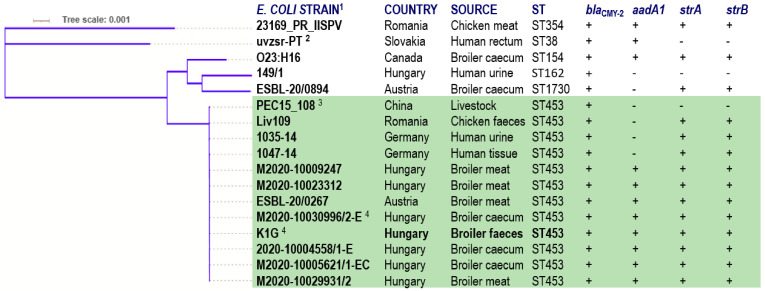
Phylogenetic tree inferred by REALPHY for selected *bla*_CMY-2_-positive *E. coli* isolates of human, animal, and food origin. ST numbers provide the 7-gene MLST sequence types. The tree scale is indicated in the upper-left corner. ^1^ The strain identifier is the NCBI SRA Run or the EnteroBase uberstrain code, provided in Section 2.10. ^2^ uvzsr-PT stands for the full isolate name of uvzsr-PT_25_00022-C06-AR_25_00002058. ^3^ Green-coloured background indicates isolates with the serotype of O23:H16. ^4^ These isolates contain a phage-like plasmid of subtype IncFIB (H89-PhagePlasmid). The + and − symbols indicate detected or undetected ARGs in the corresponding WGS data, respectively.

A phylogenetic tree of selected representative *bla*_CMY-2_-positive *E. coli* isolates of various sequence types (Figure 6) showed that O23:H16-ST453 isolates of human, broiler gut microbiome, and broiler meat origin from Hungary and other neighboring or regional European countries clustered closely together, thus suggesting a potential molecular epidemiological link between them.

## 4. Discussion

The faecal bacterial composition of the examined broiler flock from Hungary was consistent with previous studies showing that the Firmicutes were the dominant phylum in healthy broiler chickens [105,106]. At the order level, dominant taxa included Lactobacillales, Oscillospirales, and Clostridia UCG-014 (Figure 1), bacterial groups that are commonly associated with carbohydrate fermentation, short-chain fatty acid production, intestinal homeostasis, and exclusion of pathogens [107,108]. In line with our findings for the faecal bacterial compositions of the four examined roosters, earlier observations have already shown that even under carefully controlled conditions, significant variations in the composition of the microbiota can occur in the gastrointestinal tracts of individual chickens within a single experiment [109]. The relatively low abundance of Enterobacterales suggests that *Enterobacteriaceae* were minor components of the faecal microbiota and that clinically relevant MDR *E. coli* may persist in the broiler gut ecosystem even when present at low abundance.

Shotgun metagenomics indicated the presence of previously uncharacterized resistance plasmids in the analyzed broiler-associated bacterial communities, with a capability to transfer resistance across different bacterial taxa and to mediate historical or ongoing plasmid–chromosome recombination and transfer events (Figure 2 and Figure 3). The detected acquired ARGs may persist in the gut microbiome due to vertical transmission, co-selection by other environmental contaminants, and by mobile genetic elements, even without significant direct antibiotic exposure [110,111]. The individual faecal samples (CSK1–CSK4) were distinguished by partially different ARG patterns, suggesting that they likely acquired some of these antibiotic resistance determinants after hatching from the egg.

Avian pathogenic *E. coli* can be responsible for a wide range of clinical conditions, including pericarditis, perihepatitis, septicemia, intestinal inflammation, arthritis, and cellulitis. In laying hens, these bacteria can cause salpingitis, which negatively impacts both the quality and quantity of egg yields [14]. APEC is normally present in the intestinal tract of poultry, together with non-virulent *E. coli* strains, and undergoes extra-intestinal translocation only in the presence of stressors [112]. A set of specific virulence genes (*hlyF*, *ompT*, *iroN*, *iss*, *tsh*, *iroN*, *cvaC*, *iucD*, and *iutA*) has been proposed for the characterization and differentiation of APEC [82,113]. Due to similar and overlapping molecular characteristics between APEC and extra-intestinal pathogenic *E. coli* (ExPEC), it was hypothesised that APEC may act as a reservoir of virulence and resistance markers, and it may be responsible for foodborne infections in humans [114]. ExPEC encompasses *E. coli* strains responsible for human neonatal meningitis (NMEC) and uropathogenic infections (UPEC) [14,115]. The presence of APEC in poultry farms, broiler processing plants, and, consequently, in retail meat, particularly chicken, may thus be potentially associated with public health risks [81].

Complete genome sequencing of the O23:H16-ST453 *E. coli* strain K1G from the faeces of a Ross-308 rooster in Hungary revealed a chromosome carrying no acquired ARGs but multiple mutation-associated AR genes; virulence factors (including *astA* encoding heat-stable enterotoxin EAST-1, *hlyE* encoding avian *E. coli* hemolysin, *lpfA* encoding long polar fimbriae, and *iss* encoding a serum resistance lipoprotein); and a diverse set of insertion sequences, transposons, and prophages. Three K1G plasmids were also identified: pEc_K1G_A, a phage-like plasmid lacking resistance or virulence genes; pEc_K1G_B, a mosaic plasmid carrying *bla*-_TEM-1b_; key APEC-associated virulence factors, including *hlyF*, *iutA*, *ompT*, *iucD*, and *cvaC*, as well as numerous mobile elements; and plasmid pEc_K1G_C, an IncC type 1 plasmid without virulence genes but with three antibiotic resistance islands that confer the MDR phenotype of K1G (Table 3).

The pEc_K1G_A plasmid carries numerous phage structural and DNA-processing genes, suggesting that phage-mediated mobilization might be possible, whereas the absence of a canonical conjugation system or the *oriT* region implies that its conjugative transfer is unlikely. Plasmid pEc_K1G_B also lacks a complete transfer apparatus, but it contains several mobile genetic elements, including IS*26*-mediated composite transposons, a *bla*_TEM-1b_ gene, and virulence regions encoding aerobactin synthesis (*iucABCD-iutA*), *sitABCD*, colicin V, and efflux-related genes. Thus, pEc_K1G_B may serve as a reservoir for virulence and resistance determinants that can be mobilized via transposition or even recombination. Although no *oriT*-like sequence has yet been identified on the plasmid, it is still possible that it is trans-mobilizable by co-resident conjugative plasmids, for which their conjugative apparatus is related to that of the ancestors of pEc_K1G_B. The plasmid’s persistence is ensured by two different replicon types (IncFIB of the RepB family and IncFIC/IncFII of the RepA1 family), a Type I partitioning system [116,117], three toxin–antitoxin systems [71], and a putative *mok/sok* post-segregational killing system, which can provide a high degree of stability to the plasmid.

The IncC type 1 pEc_K1G_C plasmid has the greatest potential for active HGT, as it encodes a conserved broad-host range conjugative transfer system (MOB_H12_ group) (refs. [28,96]) regulated by AcaDC [37,89,118], as well as several antibiotic resistance islands (ARI-A, ARI-B, and ARI-ISEcp1) [28]. The plasmid carries clinically relevant resistance genes, including *bla*_CMY-2_, *floR*, *tetA*, *sul1*, and *aadA1* and genes of other aminoglycoside-modifying enzymes (Table 2). The large number of IS*26* and other recombination-promoting IS elements and the large-scale rearrangements identified in pEc_K1G_C relative to the canonical IncC backbone highlight the dynamic evolution of this plasmid and its ability to mediate the spread of multidrug resistance between species. Although the conjugation of pEc_K1G_C is impaired due to rearrangements, an incoming SGI1 island was capable of complementing these defects and facilitating the horizontal transfer of the pEc_K1G_C plasmid.

*E. coli* K1G, with its diverse mobile element and plasmid repertoire, can be an important vehicle in the lateral transfer of ARGs and virulence genes. The *bla*_CMY-2_ β-lactamase gene detected in plasmid pEc_K1G_C is typically found in the type 1 group of IncC plasmids. Based on our IncC plasmid sequence collection derived from GenBank, it is present in 17.8% (242) of all IncCs, of which 77.3% are type 1, 19.4% are type 1/2 hybrids, and only 1% are Type 2 and the others are non-classifiable. Notably, *bla*_CMY_-carrying plasmids consistently mediate multidrug resistance (MDR) due to their association with transposons and integrons, and the conjugative transfer of these *bla*_CMY-2_ MDR plasmids has often been linked to the IncC replicon type [119,120]. The highly conserved backbone of IncC plasmids and the fast evolution of ARIs located at specific sites of the backbone facilitate the acquisition of diverse resistance genes [94]. Ultimately, the broad host range of these plasmids likely drives the global dissemination of *bla*_CMY-2_ genes.

The examined broiler chickens possessed a diverse faecal resistome (Figure 2) and associated mobile genetic elements (Figure 3), including an *aadA1*-bearing class 1 integron and *IS1595-*family elements (Supplementary Table S4). Class 1 integrons carrying a single *aadA1* cassette (Figure 3A) have frequently been detected in poultry flocks both in Hungary and abroad [92,121]. Associated conjugation experiments indicated that most of the examined intestinal *E. coli* isolates may have served as potential reservoirs for the spread of antimicrobial resistance genes and as a source for the emergence of MDR avian pathogenic *E. coli* [121].

It has been shown that the transfer of ESBL/pAmpC *E. coli* strains across the poultry production stages is likely to be significant, as about half of the *E. coli* genotypes detected in a specific production stage likely originated from an earlier stage, including breeding farms, hatcheries, fattening farms, slaughterhouses, and retail meat [122]. For the transfer of ESBL/pAmpC-producing strains, four main routes were proposed: (1) vertical transmission from parent to offspring, (2) transmission within the hatchery, (3) horizontal transmission on farms, and (4) horizontal transmission between farms and their environment [123]. Applying strict hygiene may reduce the risk of AMR transmission; however, in several studies, even intensive cleaning and disinfection practices have not completely eliminated antibiotic-resistant isolates [122,123]. Contamination of the environment of farms (such as litter, air, dust, faeces, slurry, insects, and wildlife) by antibiotic-resistant microbes might also contribute to indirect AMR transmission between farms, houses, and subsequent flocks [124,125], even in antibiotic-free production systems. These transmission pathways may also potentially facilitate human exposure to poultry-associated *E. coli*, for example, through foodborne contamination, occupational exposure of farm, hatchery or slaughterhouse workers, and by environmental dissemination to humans via an intermediary mechanism, such as soil or water [126,127,128].

In a large-scale pan-European surveillance study, 2993 *Escherichia* spp. isolates were collected from the faeces of healthy cattle, pigs, and chickens, where 100 *Escherichia* spp. isolates (0.5% in cattle, 1.3% in pigs, and 8.0% in chickens) were cefotaxime and/or ceftazidime non-wildtype. In silico screening of these non-wildtype isolates revealed that *bla*_CMY–2_ (at 22.2%) was the third most predominant ESBL/pAmpC type. The authors also suggested that the dissemination of *E. coli* cephalosporin resistance genes in European livestock communities likely primarily happens via distinct, globally successful plasmid lineages [129]. This was suggested by the observation that plasmid backbones within different plasmid lineages were often nearly identical and were also shared by phylogenetically unrelated *E. coli* isolates from chickens, cattle, and swine. Furthermore, they showed significant similarity to plasmids of human *E. coli* isolates [129].

The phylogenetic tree constructed for selected *bla*_CMY-2_-positive *E. coli* isolates revealed that the O23:H16-ST453 isolates of human, broiler gut, and broiler meat origin from Hungary and other European countries clustered closely together, supporting a potential epidemiological link between them (Figure 6). ST453 *E. coli* has been identified in both poultry and human samples in Europe, suggesting that it can occur in multiple hosts and may potentially have a zoonotic transmission potential, although a direct transmission between poultry and humans has not yet been clearly demonstrated [130,131]. In a study performed in Galicia (Spain) on chicken and turkey meat, MDR *E. coli* isolates belonging to the clone O23:H16-B1-ST453-CC86 were also recovered. Importantly, MDR *E. coli* of human clinical origin and characterized as B1-ST453 was also reported from the same health area of Galicia, thus demonstrating the importance of a One Health approach against the cross-domain dissemination of antimicrobial resistance [132].

Although our study was conducted by analyzing a limited number of rooster broiler chickens from Hungary (*n* = 4), which is acknowledged as a limitation when interpreting its broader significance, the obtained results could be placed into a wider regional and One Health perspective by performing comparative genomics analyses of the characterized MDR *E. coli* strain K1G by use of public databases.

## 5. Conclusions

The genetic features of the MDR ST453 *E. coli* strain K1G and the reported identification of MDR K1G-like ST453 *E. coli* strains from broiler caecal, faecal, and broiler meat samples in Hungary may possibly have a One Health and food safety importance as well because of the potential spread of these isolates and their resistance plasmids to the human population through the food chain. The plasmids of *E. coli* K1G represent a combination of a host-adapted and virulence-linked IncF plasmid with a broad host range multidrug-resistance IncC plasmid and its associated MGEs (such as IS*26*, class 1 integron, and Tn*21*). Together, they form a potent platform for the evolution, maintenance, and dissemination of multidrug resistance in poultry and potentially also across the One Health domains. The identified class 1 integron carrying *aadA1* and *aac(3)-VIa* may be particularly important, as integrons are major contributors to ARG accumulation and dissemination [6,133,134]. Our findings have highlighted the complexity of factors impacting plasmid mobility and phenotypic antibiotic resistance in MDR *E. coli* strains of food animal origin.

## Figures and Tables

**Table 1:** Susceptibility of *E. coli* strain K1G against various antibiotics ^1,2^.

Antibiotic	Ampicillin	Cefotaxime	Ceftazidime	Imipenem	Meopenem	Gentamicin	Amikacin	Streptomycin	Tetracycline	Sulfamethoxazole	Trimethoprim	Nalidixic Acid	Ciprofloxacin	Levofloxacin	Colistin	Chloramphenicol
**Interpretation**	R	R	R	S	S	R	S	Non-WT	Non-WT	Non-WT	S	Non-WT	R	R	WT	Non-WT

^1^ Interpretation of in vitro testing is based on the EUCAST Clinical Breakpoint Tables v. 16.0 (valid from 1 January 2026). ^2^ Interpretation of wild-type (WT) and non-wild-type (Non-WT) phenotypes is based on the current EUCAST Epidemiological Cutoff (ECOFF) values for *E. coli* available at https://www.eucast.org/vetcast/mic-distributions-and-ecoffs/, accessed on 13 July 2026.

**Table 2:** Acquired ARGs detected in the plasmids of *E. coli* strain K1G.

Acquired ARG	Identity with the Reference (%)	Reference	Resistance Phenotype	Plasmid Localization of the ARG
*sul1*	99.89	EU780013	Sulfamethoxazole	pEc_K1G_C
*aac(3)-VIa*	100	NC_009838	Gentamicin	pEc_K1G_C
*ant(3* *″* *)-Ia (aadA1)*	99.75	X02340	Streptomycin	pEc_K1G_C
*bla_CMY-2_*	100	X91840	Ampicillin, Cefotaxime, Ceftazidime, Piperacillin+Tazobactam	pEc_K1G_C
*sul2*	100	AY034138	Sulfamethoxazole	pEc_K1G_C
*aph(3″)-Ib (strA)*	100	AF321551	Streptomycin	pEc_K1G_C
*aph(6)-Id (strB)*	100	M28829	Streptomycin	pEc_K1G_C
*tet(A)*	100	AF534183	Doxycycline, Tetracycline	pEc_K1G_C
*floR*	99.83	CP136914	Chloramphenicol, Florfenicol	pEc_K1G_C
*bla_TEM-1B_*	100	AY458016	Amoxicillin, Ampicillin, Cephalothin, Piperacillin, Ticarcillin	pEc_K1G_B

**Table 3:** The main features of the three plasmids harbored by *E. coli* strain K1G.

Plasmid Name	pEc_K1G_A	pEc_K1G_B	pEc_K1G_C
Plasmid length	113,150 bp	86,062 bp	146,632 bp
Incompatibility Group	IncF	IncF	IncC
Replicon subtype	IncFIB(H89-PhagePlasmid)	IncFIB and IncFIC(FII)	IncC Type 1
Plasmid replication initiator proteins	RepB	RepB and RepA	RepA
Generic description	Hybrid element containing both phage-related genes and an FIB replicon	*Enterobacteriaceae* mosaic plasmid	Broad host range multidrug-resistance plasmid
APEC virulence factors	None	*hlyF*, *iutA*, *ompT*, *iucD*, *cvaC*	None
Acquired ARGs identified by ResFinder	None	*bla*_TEM-1B_	*aac(3)-VIa*, *aadA1*, *aph(3″)-Ib*, *aph(6)-Id*, *bla*_CMY-2_, *floR*, *sul1*, *sul2*, *tet(A)*
Other selected plasmid- encoded proteins	Phage tail protein, Tail assembly protein, Major capsid protein	Colicin V and Colicin V Immunity protein, SitABCD hydrogen peroxide resistance	Class 1 Integrase, Disinfectant-resistance protein QacEdelta
Top GenBank BLASTN hit (coverage %; identity %)	*E. coli* strain RHB03-SO-C05 plasmid unnamed1 (94%; 99.6%)	*E. coli* strain CT265 plasmid pCT265_1 (98%; 99.9%)	*S. enterica* strain SL-312 plasmid pET8.1-IncAC2 (89%; 99.9%)
Isolation source for the top GenBank BLASTN hit	Soil sample collected from the floor of a pig farm in the United Kingdom	Charaterized from an avian host by Nanjing Agricultural University, China	Collected from a chicken host by the Public Health Agency of Canada

## Data Availability

The original contributions presented in this study are included in the article/Supplementary Materials. Further inquiries can be directed to the corresponding authors. The complete genome assembly of *E. coli* strain K1G was submitted to the NCBI Genomes database under BioProject ID PRJNA1212878 and to the *Escherichia* spp. PubMLST database with the following ID: BIGSdb_20260617125426_573644_70417. Furthermore, a genome assembly of *E. coli* strain K1G is also available in the Enterobase database with the uberstrain code ESC_JB4571AA.

## Supplementary Materials

The following supporting information can be downloaded at https://www.mdpi.com/article/10.3390/ani16152322/s1. Figure S1: The schematic map of plasmid pEc_K1G_A; Figure S2: The schematic map of plasmid pEc_K1G_B; Figure S3: The schematic map of plasmid pEc_K1G_C; Figure S4: Colony PCR test of transconjugants; Table S1: Composition of diets of broiler chickens; Table S2: List of oligonucleotides used; Table S3: Bacterial strains and plasmids used in this study; Table S4: Boiler fecal metagenomic contigs; Table S5: Mobile elements detected by IS Finder in *E. coli* strain K1G; Table S6: O23:H16 *E. coli* strains of worldwide locations; Table S7: K1G-like *E. coli* strains from Hungary.
