# Supplementary material for: Genetic Elements Associated with the Acquired Resistome of the Gut Microbiota in a Broiler Rooster Flock in Hungary
Source: Animals (Basel). 2026 Jul 29;16(15):2322. doi: 10.3390/ani16152322 (PMC13463583; doi:10.3390/ani16152322)
Supplement: Supplementary file 1 [file animals-16-02322-s001.zip › SUPPLEMENTARY MATERIALS_20260727_REVIEWED.pdf]

SUPPLEMENTARY MATERIALS

Genetic elements associated with the acquired resistome of broiler gut microbiota in Hungary

János Kiss, Balázs Libisch, Chioma Lilian Ozoaduche, Hedvig Fébel, Geertrui Rasschaert, Ellen Lambrecht, Marc Heyndrickx, Mónika Szabó, Tibor Keresztény, Katalin Posta and Ferenc Olasz.

SUPPLEMENTARY FIGURE S1. THE SCHEMATIC MAP OF PLASMID pEc_K1G_A.

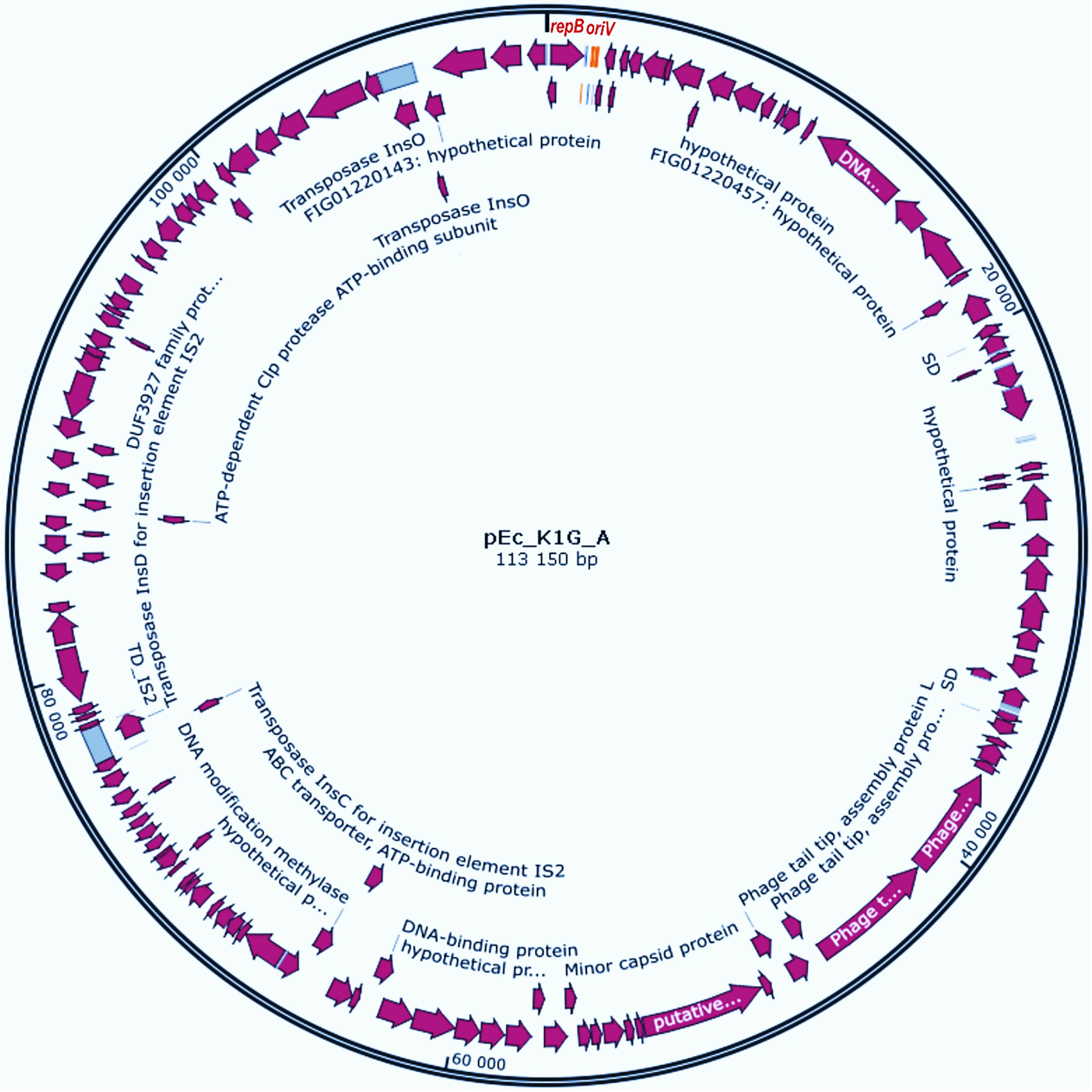

Supplementary Figure S1. The schematic map of pEc_K1G_A. Annotated ORFs are shown as purple arrows, IS elements are marked by light blue boxes. *RepB* and *oriV* are indicated in red.

SUPPLEMENTARY FIGURE S2. THE SCHEMATIC MAP OF PLASMID pEc_K1G_B.

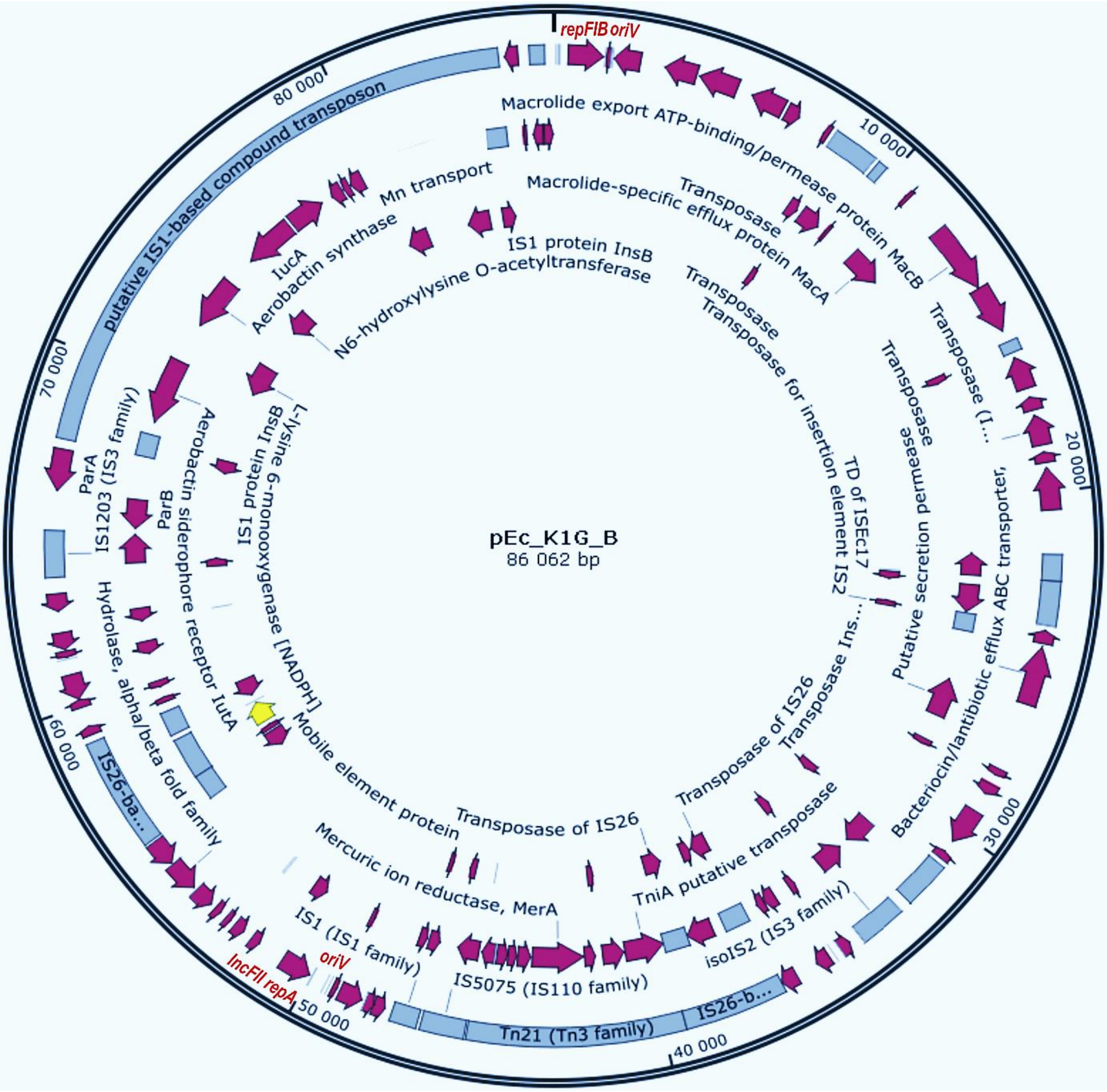

Supplementary Figure S2. The schematic map of pEc_K1G_B. *RepFIB*, *IncFII repA* and the predicted *oriV* regions are indicated in red. Mobile elements and transposon-derived regions are shown as light blue boxes. ARG is highlighted in yellow.

SUPPLEMENTARY FIGURE S3. THE SCHEMATIC MAP OF PLASMID pEc_K1G_C.

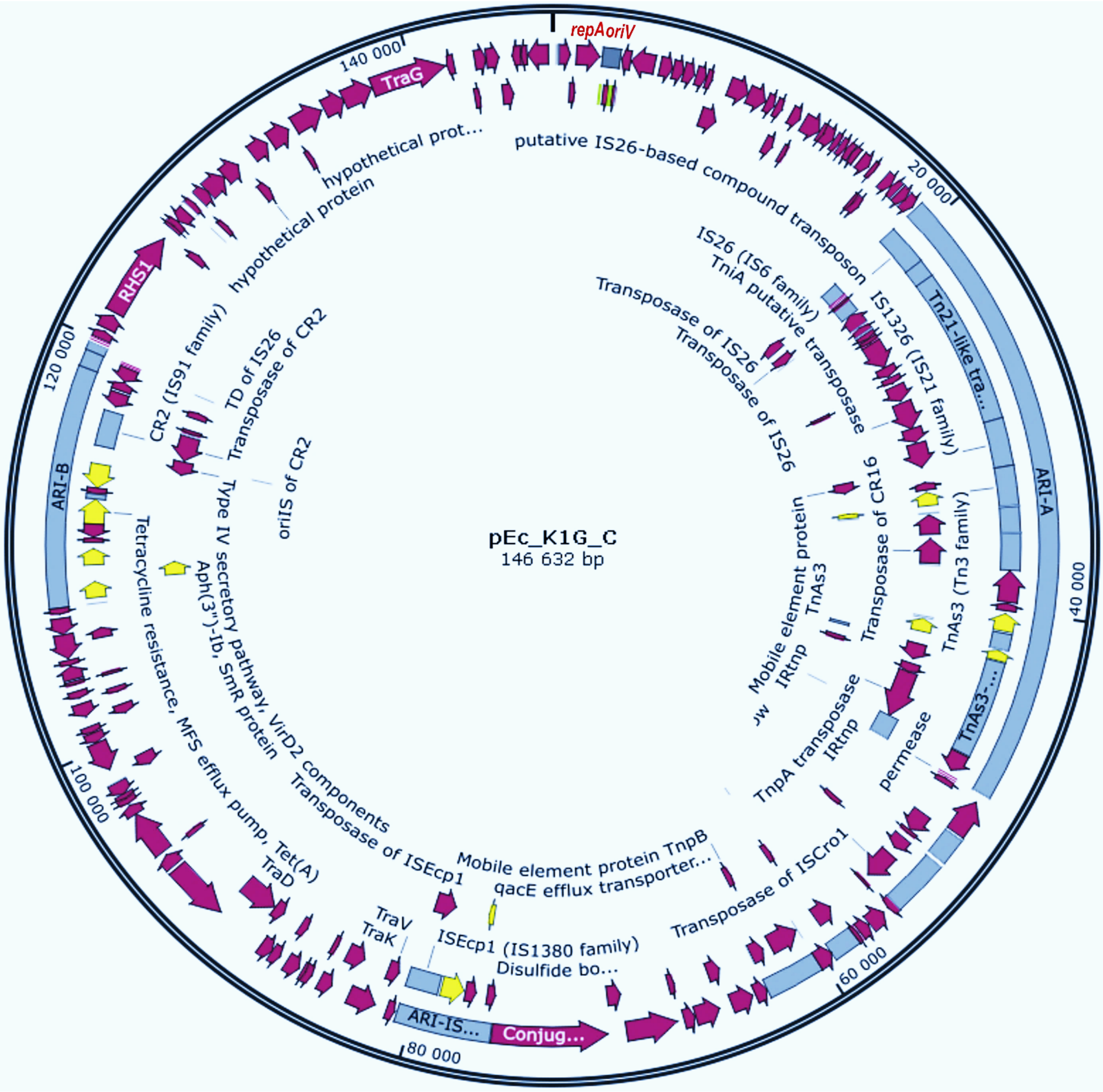

Supplementary Figure S3. The schematic map of pEc_K1G_C. *RepA* and *oriV* are indicated in red. Mobile elements and transposon-derived regions are shown as light blue boxes. ARGs are highlighted in yellow.

SUPPLEMENTARY FIGURE S4. COLONY PCR TEST OF TRANSCONJUGANTS

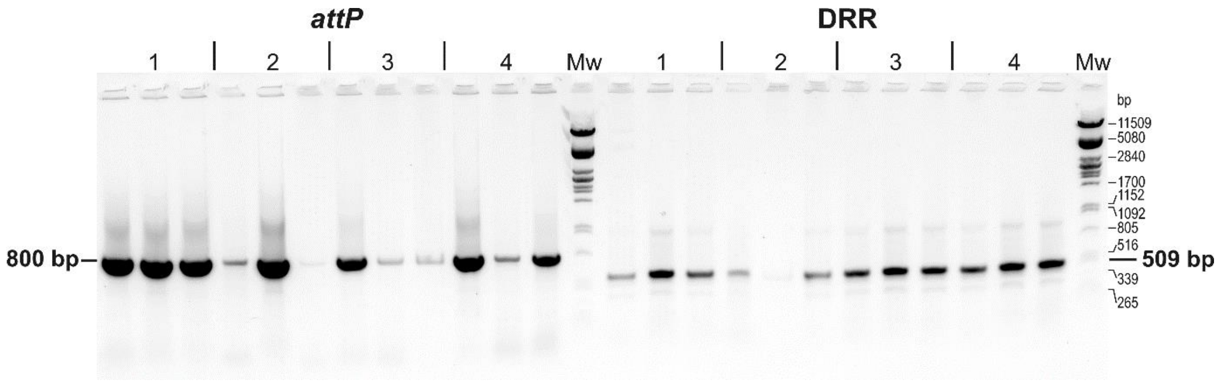

Supplementary Figure S4. Colony PCR test of TG1Rif::SGI1-C$^{\Delta 5}$/pEc_K1G_C transconjugants. Three Rif^{R}Km^{R}Cm^{R} colonies were selected from four independent TG1Nal::SGI1-C$^{\Delta P5}$/R55$^{\Delta Tn\mathit{6187}}$ × TG1Rif/pEc_K1G_C matings for amplification of *attP* and DRR confirming the presence of SGI1-C$^{\Delta P5}$ Size of amplicons is indicated. Mw – λ DNA digested with *Pst*I.

SUPPLEMENTARY TABLE S1. COMPOSITION OF DIETS OF BROILER CHICKENS

Ingredient	Composition of diets (%) in different growing phases		
	Starter	Grower	Finisher
corn	50.7	55	59.7
extracted soybean meal	37	33	28
full-fat soybean	6	6	6
oil	2	2.32	2.5
limestone	1.6	1.25	1.34
monocalcium phosphate (MCP)	1.4	1.33	1.35
NaCl	0.4	0.4	0.4
L-lysine HCl	0.12	0.05	0.06
DL -methionine	0.19	0.16	0.14
L-threonine	0.03	0	0
premix	0.5	0.5	0.5
butylated hydroxy toluene (BHT)	0.01	0.014	0.014

SUPPLEMENTARY TABLE S2. LIST OF OLIGONUCLEOTIDES USED

Primer	Sequence (5′-3′) [a]	Reference
amprevXP	AATCTAGACTGCAGGGTCTGACAGTTACCAATGC	[36]
BXhNdtac	CGGGATCCCTCGAGCATATGTGTTTCCTGTGTGAAATTGT	[37]
attsgi1rev	GCTCTAGAGCGGCCGCAAATGTGAATCGAATCACAATCG	[38]
LJ2	AGCTGCAGCGGCCGCAAGTTTTACTCTGTCTTCCAG	[38]
RJ2	AGCTGCAGCGGCCGCTCGAAGAGGTAGAGCAG	[38]
27F	AGAGTTTGATCCTGGCTCAG	[6]
1492R	GGTTACCTTGTTACGACTT	[6]
16S-F	TCGTCGGCAGCGTCAGATGTGTATAAGAGACAGCCTACGG GNGGCWGCAG	[6]
16S-R	GTCTCGTGGGCTCGGAGATGTGTATAAGAGACAGGACTAC HVGGTATCTAATCC	[6]
poxfor37015	CACCACCAAAAGCACCACACCC	this work
poxrev37376	GATATACAGGTTCACCTTAGCC	this work

[a] Restriction sites in non-annealing 5’ends are underlined.

SUPPLEMENTARY TABLE S3. BACTERIAL STRAINS AND PLASMIDS USED IN THIS STUDY

E. coli strains	Relevant features	References
TG2/R55$^{\Delta Tn6187}$	derivative of TG2 strain [56] containing the IncC plasmid R55$^{\Delta Tn6187}$, Flo^R, Cm^R. Tc^R	this work
TG1	*supE hsd*Δ5 *thi* Δ(*lac-proAB*) F'[*traD36 proAB+ lacIq lacZ*ΔM15]	[57]
TG1Rif	Rif^R derivative of TG1	this work
TG1Nal	Nal^R derivative of TG1	[38]
Ec_K1G	Original strain isolated from chicken, Nal^R, Ap^R, Cm^R, Flo^R, Sm^R, Sp^R, Tc^R, Sul^R, Gm^R, Km^S, Rif^S.	this work
Ec_K1G/pGMY6	Ec_K1G derivative containing pGMY6 (p15A, Km^R) expressing the SGI1-derived FlhDC-family activator SgaDC, Nal^R, Ap^R, Cm^R, Flo^R, Sm^R, Sp^R, Tc^R, Sul^R, Gm^R, Km^R, Rif^S.	this work
Ec_K1G/pJKI888	Ec_K1G derivative containing pJKI888 (p15A, Km^R) expressing the R55-derived FlhDC-family activator AcaDC, Nal^R, Ap^R, Cm^R, Flo^R, Sm^R, Sp^R, Tc^R, Sul^R, Gm^R, Km^R, Rif^S.	this work
TG1Nal::SGI1-C$^{\Delta P5}$	TG1Nal strain containing SGI1-C with the *aph(3')-I* gene inserted upstream of orf S022, Nal^R, Sm^R, Sp^R, Sul^R, Km^R	[58]
TG1Rif/pEc_K1G_C	TG1Rif transconjugant containing the pEc_K1G_C plasmid, Rif^R, Ap^R, Cm^R, Flo^R, Sm^R, Sp^R, Tc^R, Sul^R, Gm^R, Nal^S.	this work
TG1Rif::SGI1-C$^{\Delta P5}$/pEc_K1G_C	TG1Rif strain containing chromosomally integrated SGI1-C$^{\Delta P5}$ and pEc_K1G_C plasmid, Rif^R, Ap^R, Cm^R, Flo^R, Sm^R, Sp^R, Tc^R, Sul^R, Gm^R, Km^R, Nal^S.	this work

Plasmids	Relevant features	
pGMY6	pJKI391 derivative containing the *sgaDC* genes (S007-S006) of SGI1 under the control of P_{tac} promoter, Km^R	[59]
pJKI888	pJKI391 derivative containing the *acaCD* genes of R55 under the control of P_{tac} promoter, Km^R	[37]
R55$^{\Delta Tn6187}$	The Tn*6187*-deleted derivative of the IncC Type 2 plasmid R55 [60], tra+, Cm^R, Flo^R, Sul^R, Ap^S, Km^S, Gm^S	[36]

SUPPLEMENTARY TABLE S4. BOILER FECAL METAGENOMIC CONTIGS

Characteristics of shotgun metagenomic contigs	Contig A	Contig B	Contig C	Contig D	Contig E
Broiler feces sample	CSK1	CSK1	CSK2	CSK3	CSK4
Contig identifier	k141 263755	k141 177008	k141 84069	k141 136742	k141 35821
Contig Length (bp)	17156	79693	113243	7075	2855
Approximate k-mer coverage	31.72	14.92	26.36	19.00	14.00
GC content (%)	56.14	46.25	34.61	34.52	31.45
Classification	Proteobacteria	Bacteroidota	*Lactobacillus*	Firmicutes	Bacteroidota
Genetic elements	Class 1 Integron	IS1595 IS Family	ISL3 IS Family	ARGs derived from a putative Tn	IS1595 IS Family
Detected acquired ARGs	*tet(C), ant(3")-Ia, sul1*	*mef(A), aph(6)-Id, aph(3")-Ib, sul2*	*lnu(A), erm(T)*	*lnu(B), lsa(E), ant(6)-Ia*	*bla* $_{OXA-347}$ *, ermF*

SUPPLEMENTARY TABLE S5. MOBILE ELEMENTS DETECTED BY IS FINDER IN *E. COLI* STRAIN K1G

This Table S5 is included as a separate Excel sheet.

SUPPLEMENTARY TABLE S6. O23:H16 *E. COLI* STRAINS OF WORLDWIDE LOCATIONS

Name	Source Niche	Source Type	Collection Year	Continent	Country	Region	Serotype	Sequence Type	ST Complex	adk	fumC	gyrB	icd	mdh	purA	recA	Aminoglycoside resistance	ESBLs	Macrolide resistance	Penicllin resistance	Phenicol resistance	Quinolone resistance	Sulfonamide resistance	Tetracycline resistance	Trimethoprim resistance	Other Classes
LA3	Environment	Hospital wastewater	2024	Asia	Philippines	Laguna	O23:H16	ST453	ST86 Cplx	99	6	33	33	24	8	7	*aadA1.aph(6)Id.aac(3)IId.aph(3')Ia.aph(3'')Ib*	*blaOXA10.blaCTXM55*		*blaTEM1*	*floR.cmlA5*	*qnrS1.gyrA_D87N.gyrA_S83L.parC_S80I*	*sul1.sul2*		*dfrA14*	*Rifamycin:arr2:Lincosamide:lnu(F)*
EC.IT5	Poultry	Avian	2017	South America	Brazil	Minas Gerais	O23:H16	ST8129	ST86 Cplx	99	6	33	33	24	607	7		*blaCTXM8.blaCTXM55*		*blaTEM*				*tet(B)*		
PEC13_037	Livestock	Livestock	2013	Asia	China	Shanghai	O23:H16	ST453	ST86 Cplx	99	6	33	33	24	8	7	*aadA1.aadA2.rmtB1.aac(3)IId.aph(3')Ia.aph(3')IIa*	*blaCTXM55*		*blaTEM*	*floR.cmlA1*	*gyrA_S83L.parC_S80I*	*sul2.sul3*	*tet(A).tet(M)*	*dfrA12.dfrA14*	*Bleomycin:ble:Lincosamide:lnu(F)*
PEC15_108	Livestock	Livestock	2015	Asia	China	Shanghai	O23:H16	ST453	ST86 Cplx	99	6	33	33	24	8	7	*aadA1.aadA2.aac(3)IId*	*blaCMY2.blaCTXM55*	*mef(B)*	*blaTEM*	*floR.cmlA1*	*qnrS1.gyrA_D87N.gyrA_S83L.parC_S80I*	*sul2.sul3*	*tet(A).tet(M)*	*dfrA12*	*Bleomycin:bleO:Lincosamide:lnu(F)*
O23:H16	Poultry	Broiler chickens	2006	North America	Canada	British Columbia	O23:H16	ST154		6	6	5	10	9	8	6	*aadA25.aph(6)Id.aac(3)VIa.aph(3'')Ib*	*blaCMY2*			*floR*		*sul1.sul2*	*tet(A)*		
**K1G**	**Poultry**	**Broiler chicken**	**2021**	**Europe**	**Hungary**	**Herceghalom**	**O23:H16**	**ST453**	**ST86 Cplx**	**99**	**6**	**33**	**33**	**24**	**8**	**7**	***aadA1,aph(6)Id,aac(3)VIa,aph(3'')Ib***	***blaCMY2***		***blaTEM1***	***floR***	***gyrA_D87N,gyrA_S83L,parC_S80I***	***sul1,sul2***	***tet(A)***		
ECC296	Human	Human urine	2017	Europe	France	Occitanie	O23:H16	ST453	ST86 Cplx	99	6	33	33	24	8	7				*blaTEM1*		*gyrA_D87N.gyrA_S83L.parC_S80I*	*sul2*	*tet(A)*	*dfrA1*	
KSU-3E	Environment	Water/River Cistern	2021	North America	United States	Kansas	O23:H16	ST453	ST86 Cplx	99	6	33	33	24	8	7	*aph(6)Id.aph(3'')Ib*							*tet(A)*		
KSU-9E	Environment	Water/River Cistern	2021	North America	United States	Kansas	O23:H16	ST453	ST86 Cplx	99	6	33	33	24	8	7	*aph(6)Id.aph(3'')Ib*							*tet(A)*		
KSU-10E	Environment	Water/River Cistern	2021	North America	United States	Kansas	O23:H16	ST453	ST86 Cplx	99	6	33	33	24	8	7	*aph(6)Id.aph(3'')Ib*							*tet(A)*		
SAMEA112624678	Poultry	Chicken caecal	2014	Europe	France	N/A	O23:H16	ST13418	ST86 Cplx	1510	6	33	33	24	8	7	*aadA1.aadA2.aph(6)Id.aph(3'')Ib*				*catA1.cmlA1*		*sul1.sul3*	*tet(A)*	*dfrA1*	
24MA01PC02-EC	Livestock	Swine pork	2024	North America	United States	Massachusetts	O23:H16	ST453	ST86 Cplx	99	6	33	33	24	8	7										
PNUSAE175644	Wild Animal	Feces	2011	North America	United States	Pennsylvania	O23:H16	ST947	ST469 Cplx	6	65	5	1	9	13	4										
24PA09CB01-EC	Poultry	Chicken Thighs	2024	North America	United States	N/A	O23:H16	ST453	ST86 Cplx	99	6	33	33	24	8	7	*aadA1*							*tet(A)*		
PNUSAE207657	Human	Human Sool	2025	North America	United States	N/A	O23:H16	ST6188		6	19	5	18	11	7	7										
0.1065	Poultry	Turkey	2000	North America	United States	Indiana	O23:H16	ST453	ST86 Cplx	99	6	33	33	24	8	7	*aadA1.aac(3)VIa*						*sul1*			
25SC10CB04-EC	Poultry	Chicken	2025	North America	United States	South Carolina	O23:H16	ST453	ST86 Cplx	99	6	33	33	24	8	7								*tet(B)*		
AMC1424	Livestock	Bovine Fecal composite, Dairy farm	2023	North America	Canada	British Columbia	O23:H16	ST336	ST278 Cplx	9	4	33	18	11	8	6										
AMC1422	Livestock	Bovine Fecal composite, Dairy farm	2023	North America	Canada	British Columbia	O23:H16	ST336	ST278 Cplx	9	4	33	18	11	8	6										
AMC1420	Livestock	Bovine Fecal composite, Dairy farm	2023	North America	Canada	British Columbia	O23:H16	ST336	ST278 Cplx	9	4	33	18	11	8	6										
PEC22_064_rep	Livestock	Livestock	2022	Asia	China	Shanghai	O23:H16	ST453	ST86 Cplx	99	6	33	33	24	8	7				*blaTEM135*	*floR*	*qnrS1*		*tet(A)*		
PEC23_088_rep	Livestock	Livestock	2023	Asia	China	Shanghai	O23:H16	ST453	ST86 Cplx	99	6	33	33	24	8	7	*aadA2*								*dfrA12*	
1765221	Human	Human	2025	Europe	United Kingdom	N/A	O23:H16	ST453	ST86 Cplx	99	6	33	33	24	8	7	*aph(6)Id.aac(3)IId.aph(3'')Ib*			*blaTEM1*	*floR*		*sul2*	*tet(A)*		

SUPPLEMENTARY TABLE S7. K1G-LIKE *E. COLI* STRAINS FROM HUNGARY

Strain Name	Country	Source Niche	Source Details	Collection Day	NCBI BioProject	NCBI SRA	NCBI Sample ID	ST	Aminoglycoside resistance	ESBL	Penicllin resistance	Phenicol resistance	Quinolone resistance	Sulfon-amide resistance	Tetra-cycline resistance
E. coli 2020-10004558/1-E	Hungary	Poultry	broiler caecum	2020.01.29	PRJEB70301	ERR14842157	SAMEA117965319	ST453	*aadA1.aph(6)-Id.aac(3)-VIa.aph(3'')-Ib*	bla_{CMY-2}	bla_{TEM-1}	*floR*	gyrA_D87N,gyrA_S83L,parC_S80I	*sul1.sul2*	*tet(A)*
E. coli M2020-10005621/1-EC	Hungary	Poultry	broiler caecum	2020.02.03	PRJEB70301	ERR14842160	SAMEA117965322	ST453	*aadA1.aph(6)-Id.aac(3)-VIa.aph(3'')-Ib*	bla_{CMY-2}	bla_{TEM-1}	*floR*	gyrA_D87N,gyrA_S83L,parC_S80I	*sul1.sul2*	*tet(A)*
E. coli M2020-10009247	Hungary	Food	broiler meat	2020.02.24	PRJEB70301	ERR14842161	SAMEA117965323	ST453	*aadA1.aph(6)-Id.aac(3)-VIa.aph(3'')-Ib*	bla_{CMY-2}	bla_{TEM-1}	*floR*	gyrA_D87N,gyrA_S83L,parC_S80I	*sul1.sul2*	*tet(A)*
E. coli M2020-10023312	Hungary	Food	broiler meat	2020.05.06	PRJEB70301	ERR14842165	SAMEA117965326	ST453	*aadA1.aph(6)-Id.aac(3)-VIa.aph(3'')-Ib*	bla_{CMY-2}	bla_{TEM-1}	*floR*	gyrA_D87N,gyrA_S83L,parC_S80I	*sul1.sul2*	*tet(A)*
E. coli M2020-10029931/2	Hungary	Food	broiler meat	2020.06.02	PRJEB70301	ERR14842171	SAMEA117965330	ST453	*aadA1.aph(6)-Id.aac(3)-VIa.aph(3'')-Ib*	bla_{CMY-2}	bla_{TEM-1}	*floR*	gyrA_D87N,gyrA_S83L,parC_S80I	*sul1.sul2*	*tet(A)*
E. coli M2020-10030996/2-E	Hungary	Poultry	broiler caecum	2020.06.09	PRJEB70301	ERR14842172	SAMEA117965331	ST453	*aadA1.aph(6)-Id.aac(3)-VIa.aph(3'')-Ib*	bla_{CMY-2}	bla_{TEM-1}	*floR*	gyrA_D87N,gyrA_S83L,parC_S80I	*sul1.sul2*	*tet(A)*
***E. coli* K1G**	**Hungary**	**Poultry**	**broiler feces**	**2021.07.21**	**PRJNA1212878**	**SRR28552532**	**SAMN46312186**	**ST453**	***aadA1,aph(6)-Id,aac(3)-VIa,aph(3'')-Ib***	$\mathbf{bla_{CMY-2}}$	$\mathbf{bla_{TEM-1}}$	***floR***	**gyrA_D87N,gyrA_S83L,parC_S80I**	***sul1,sul2***	***tet(A)***
